# Supplementary material for: mRNA-LNP vaccine strategies: Effects of adjuvants on non-parenchymal liver cells and tolerance
Source: Mol Ther Methods Clin Dev. 2025 Feb 4;33(1):101427. doi: 10.1016/j.omtm.2025.101427 (PMC11872076; doi:10.1016/j.omtm.2025.101427)
Supplement: Document S1. Figures S1–S10 and Tables S1 and S2 [file mmc1.pdf]

OMTM, Volume *33*

Supplemental information

mRNA-LNP vaccine strategies: Effects of adjuvants on non-parenchymal liver cells and tolerance

Malin Svensson, María José Limeres, Yanira Zeyn, Rocio C. Gambaro, German A. Islan, Ignacio Rivero Berti, Silvia Fraude-El Ghazi, Leah Pretsch, Katja Hilbert, Paul Schneider, Leonard Kaps, Matthias Bros, Stephan Gehring, and Maximiliano L. Cacicedo

Supplemental Material

Table S1: Characterization of Genvoy-ILM^{TM} LNPs by dynamic light scattering (DLS) and quantification of encapsulation efficiency (EE) by modified Ribogreen® assay.

Formulation	Mean size (nm)	PDI index	Z potential (mV)	EE (%)
OVA mRNA-LNPs	93.4 ± 0.7	0.051 ± 0.009	-2.7 ± 0.5	85.0 ± 2.0
Luc mRNA-LNPs	102.4 ± 2.1	0.063 ± 0.010	-2.0 ± 0.5	93.1 ± 1.5

PDI: polydispersity index

Table S2: Inventory of antibodies applied for flow cytometry analysis.

Marker	Fluorochrome	Clone	Reference	Dilution
Characterization of NPC subpopulations				
F4/80	Alexa Fluor 488	BM8	123120	1/50
CD32b	PE	AT130-2	12-0321-82	1/50
CD11c	PE-Cy7	N418	117318	1/80
CD45	eFluor 506	30-F11	69-0451-82	1/40
CD80	Super Bright 436	16-10A1	62-0801-82	1/333
CD86	APC	GL-1	105012	1/133
Viability dye	7-AAD	-	559925	1/25
Activation state of the NPC subpopulations (immunization study)				
F4/80	Alexa Fluor 488	BM8	123120	1/50
CD32b	PE	AT130-2	12-0321-82	1/50
CD11c	BV650	N418	117339	1/80
CD45	BUV395	30-F11	363-0451-82	1/200
CD80	BUV563	16-10A1	741272	1/40
CD86	BV421	GL-1	105032	1/80
MHC-II (I-A/I-E)	APC-eFluor 780	M5/114.15.2	47-5321-82	1/320

Viability dye	7-AAD	-	559925	1/25
T cell stimulation by intracellular cytokine staining				
CD3	eFluor 506	17A2	69-0032-82	1/66
CD4	FITC	L3T4	553047	1/833
CD8α	Super Bright 436	53-6.7	62-0081-82	1/125
IL-2	PE	JES6-5H4	554428	1/80
IFN-γ	APC	XMG1.2	554413	1/166
TNF-α	PE-Cy7	MP6-XT22	25-7321-82	1/166
Viability dye	L/D Fix Near-IR	-	L34975	0.5:1000
T cell proliferation and Treg response by intranuclear staining				
CD3	eFluor 506	17A2	69-0032-82	1/66
CD4	FITC	L3T4	553047	1/833
CD8α	Super Bright 436	53-6.7	62-0081-82	1/125
CD25	PE-Cy7	PC61.5	25-0251-82	1/200
FoxP3	APC	FJK-16s	17-5773-82	1/20
Ki-67	PE	REA183	130-120-417	1/50
Viability dye	L/D Fix Near-IR	-	L34975	0.5:1000

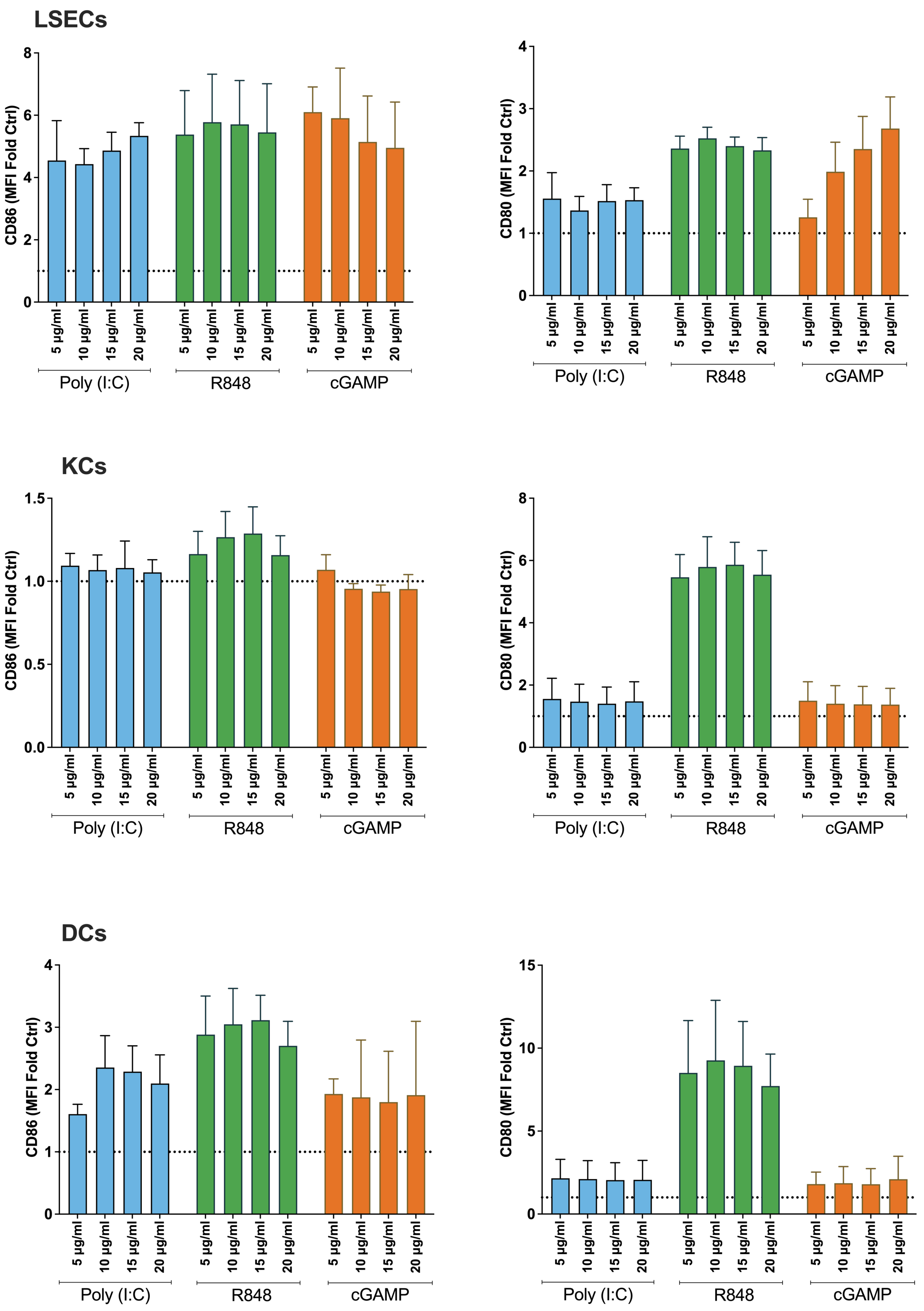

Figure S1. Adjuvants stimulate the expression of CD86 and CD80 by NPC subpopulations. Dose titration was performed in NPC cultures with 5 to 20 µg/mL R848, Poly I:C or cGAMP. CD86 and CD80 expression was evaluated by flow cytometry in Kupffer cells (KCs), dendritic cells (DCs) and Liver sinusoidal endothelial cells (LSECs). Data are the means ± SEM (n = 3).

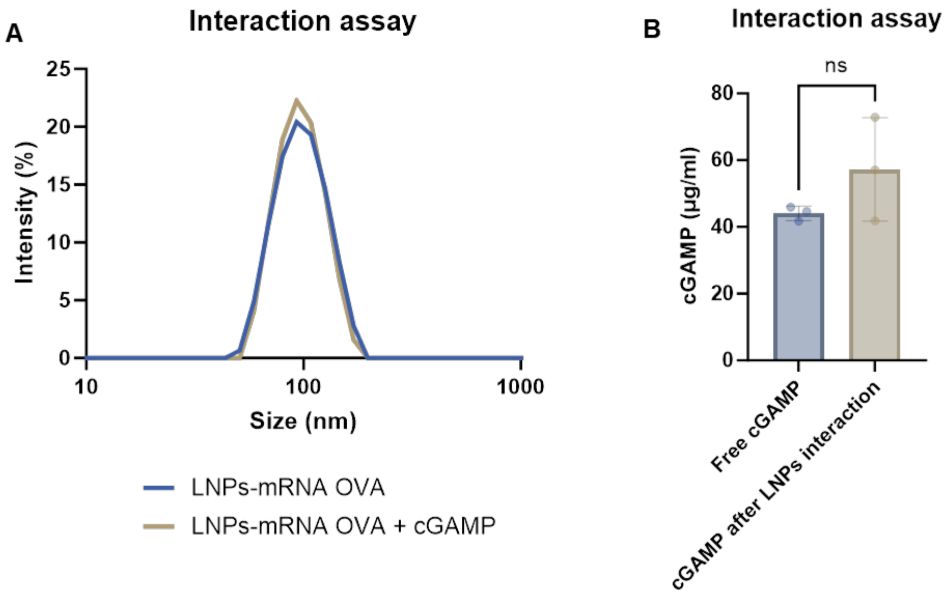

Figure S2. Interaction assay between LNPs-mRNA OVA and cGAMP. 60 µL of LNPs-mRNA OVA containing 7 µg of mRNA were mixed with 10 µL (5 µg) of cGAMP. The volume was adjusted to 100 µL with PBS. Controls of LNPs without cGAMP and free cGAMP were performed. The mixture was left interacting for 10 min at 25°C. After incubation, the mean size of the LNPs was measured by DLS. Then, 500 µL of the mixture were transferred to a centrifugal device (Microcon® centrifugal filters 100 kDa, Merck Millipore, Billerica, MA, USA) and the concentration of cGAMP was determined in the filtrate by ELISA.

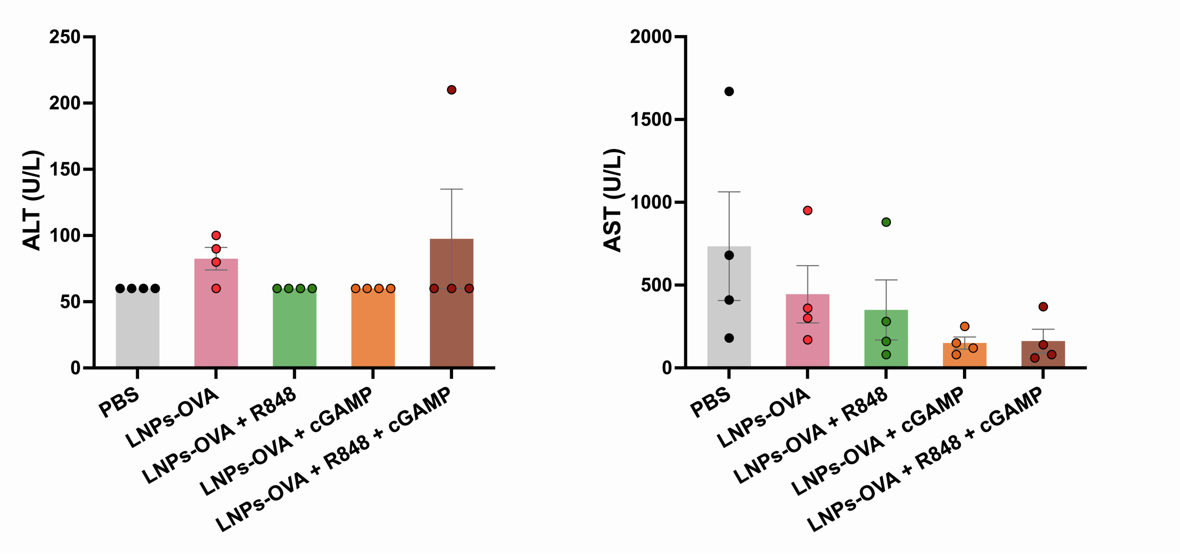

Figure S3. Serum alanine transaminase (ALT) and aspartate transaminase (AST) at termination of the immunization study. Data are mean ± SEM (n = 4). Non-significant differences were found between groups (one-way ANOVA, Tukey´s multiple comparison test).

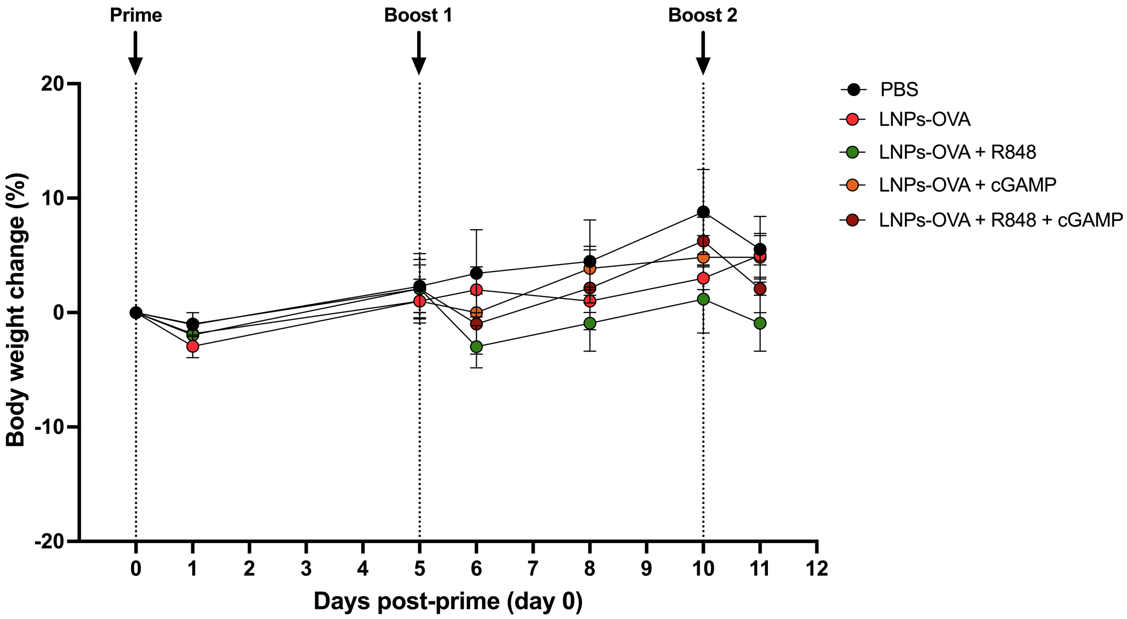

Figure S4. Mice body weight change of the immunization study. The reactogenicity of the LNP formulations plus adjuvants was measured by calculation of the body weight change of the immunized mice.

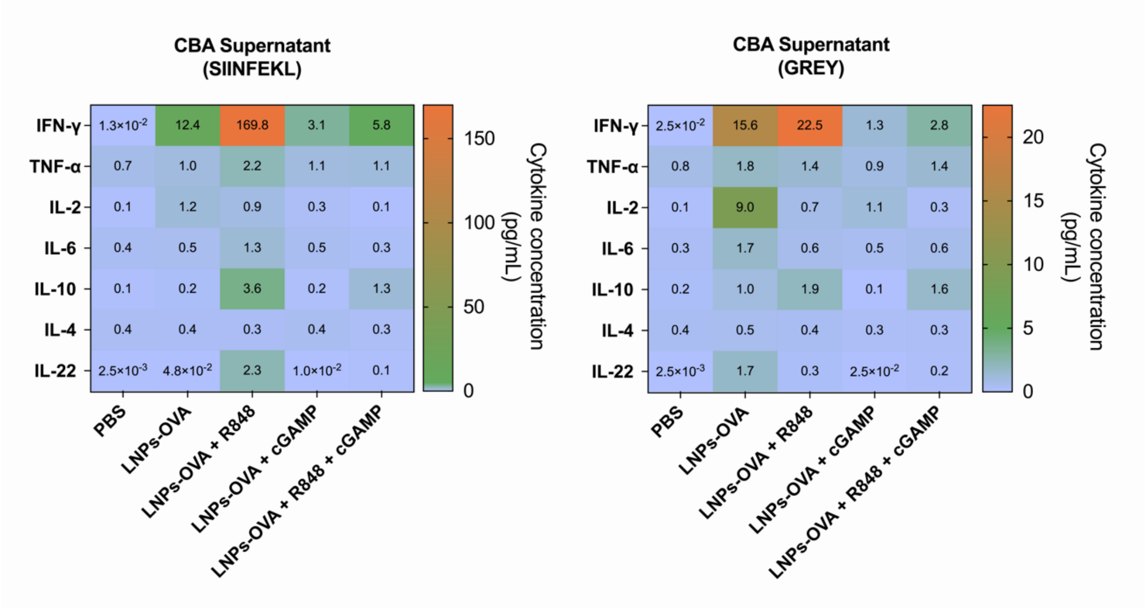

Figure S5. Cytokine quantification in supernatant of 24h-peptide-stimulated splenocytes. Single-cell spleen suspensions from immunized mice were seeded and stimulated with SIINFEKL and GREY peptides for 24h. Supernatants were collected, and the cytokine concentrations were determined using the LEGENDplex mouse Th Cytokine Panel (BioLegend).

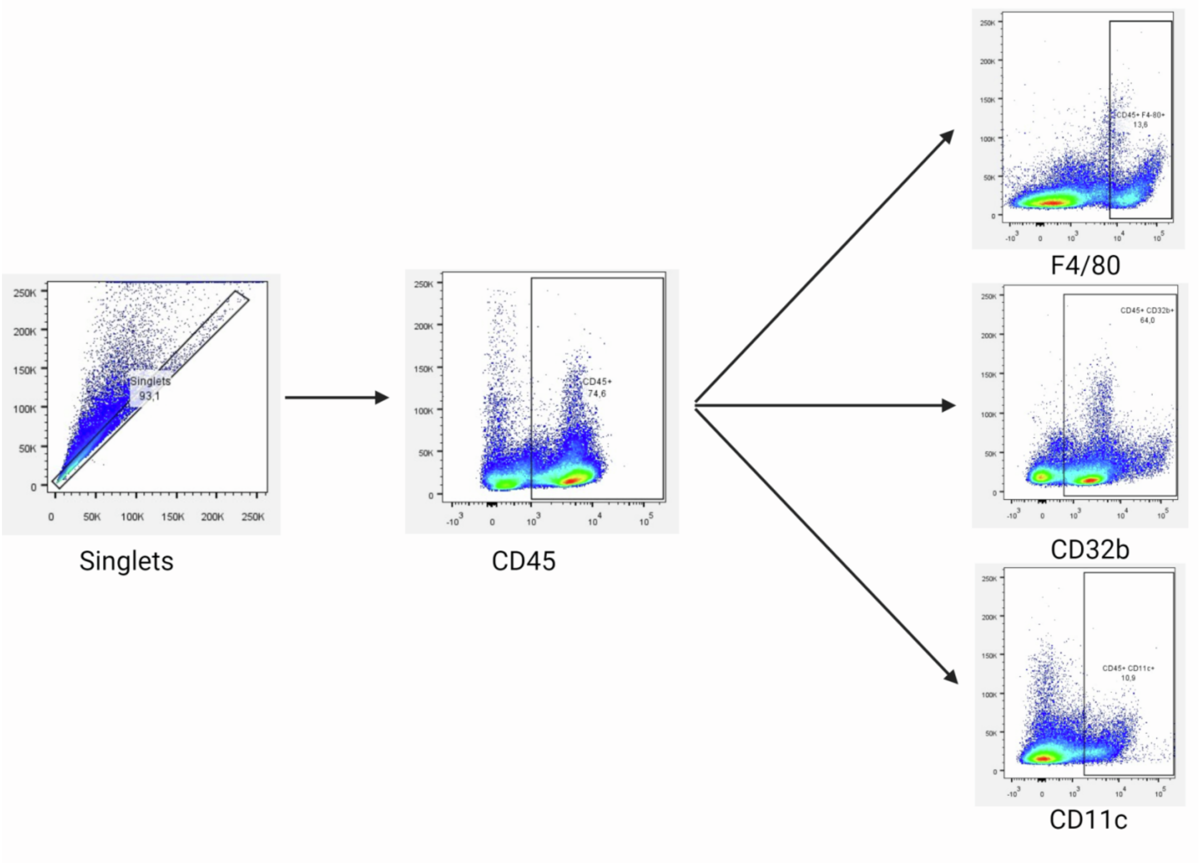

Figure S6. Gating strategy for the different viable NPC subpopulations. Kupffer cells (KCs): $CD45^{+}$ $F4/80^{+}$; Liver sinusoidal endothelial cells (LSECs): $CD45^{+}$ $CD32b^{+}$; Dendritic cells (DCs): $CD45^{+}$ $CD11c^{+}$.

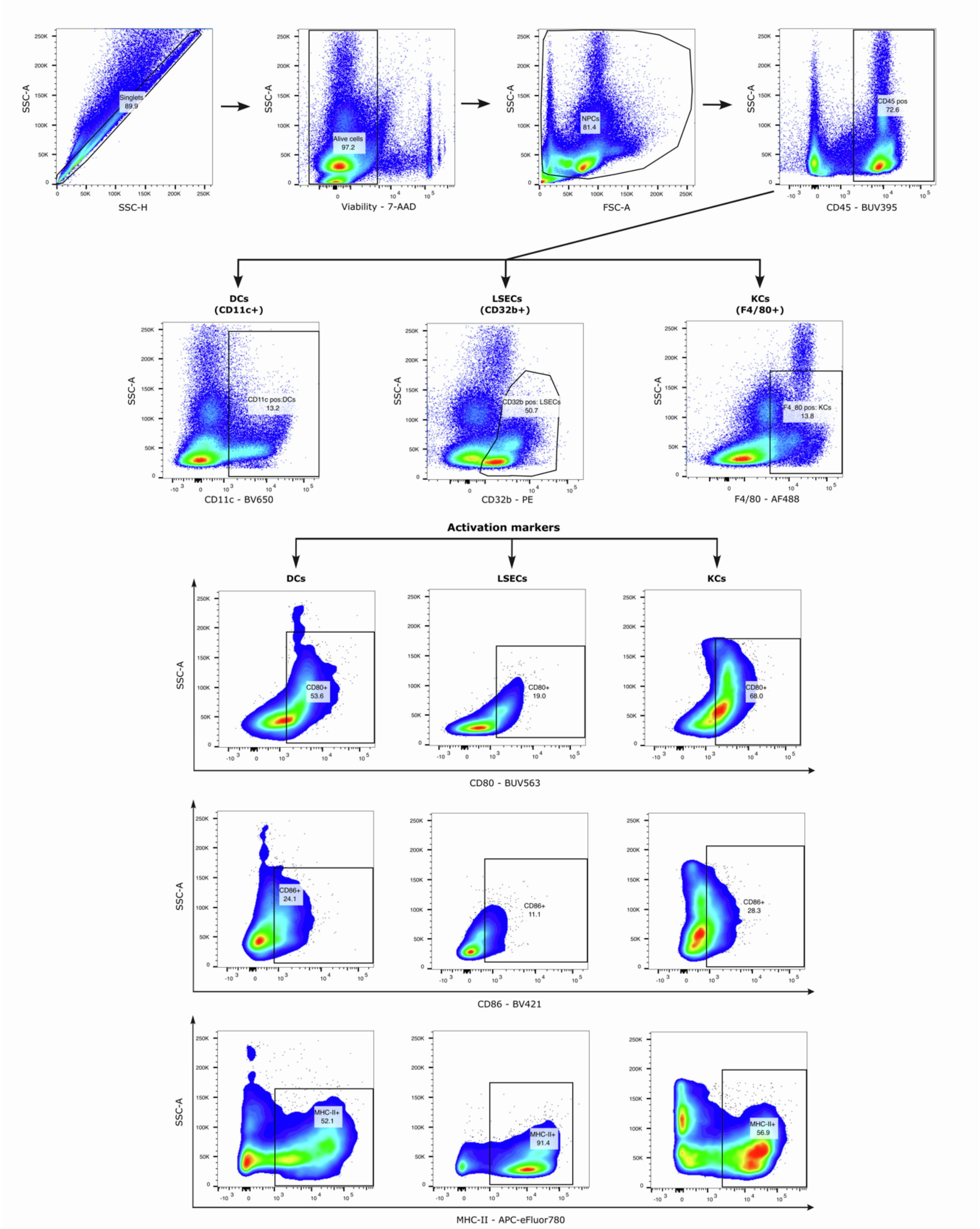

Figure S7. Gating strategy for the activation state (CD80, CD86 and MHC-II) of the different NPC subpopulations of the immunization study. Dendritic cells (DCs): CD45^{+} CD11c^{+}; Liver sinusoidal endothelial cells (LSECs): CD45^{+} CD32b^{+}; Kupffer cells (KCs): CD45^{+} F4/80^{+}.

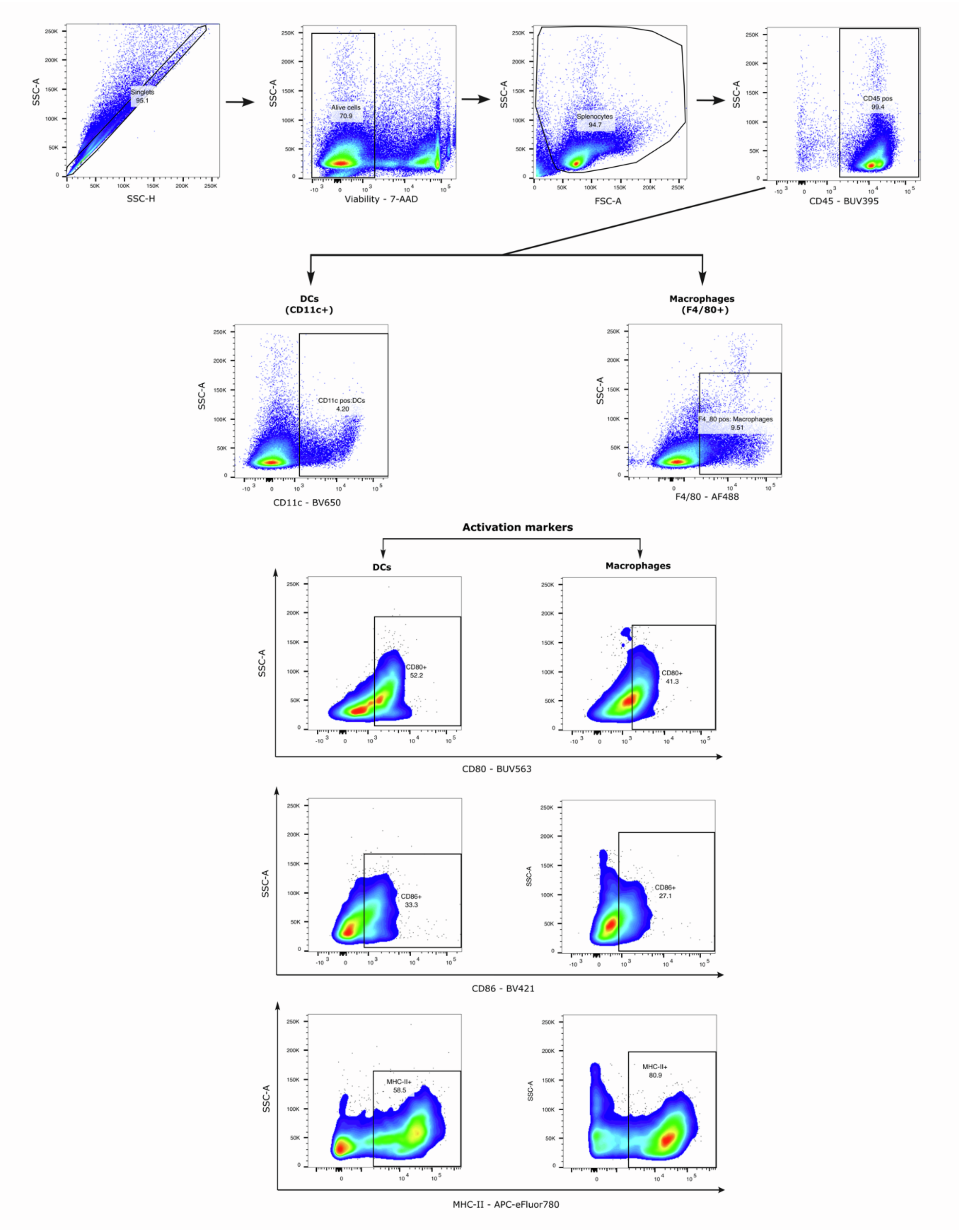

Figure S8. Gating strategy for the activation state (CD80, CD86 and MHC-II) of the different splenocyte subpopulations of the immunization study. Dendritic cells (DCs): CD45$^+$ CD11c$^+$; macrophages: CD45$^+$ F4/80$^+$.

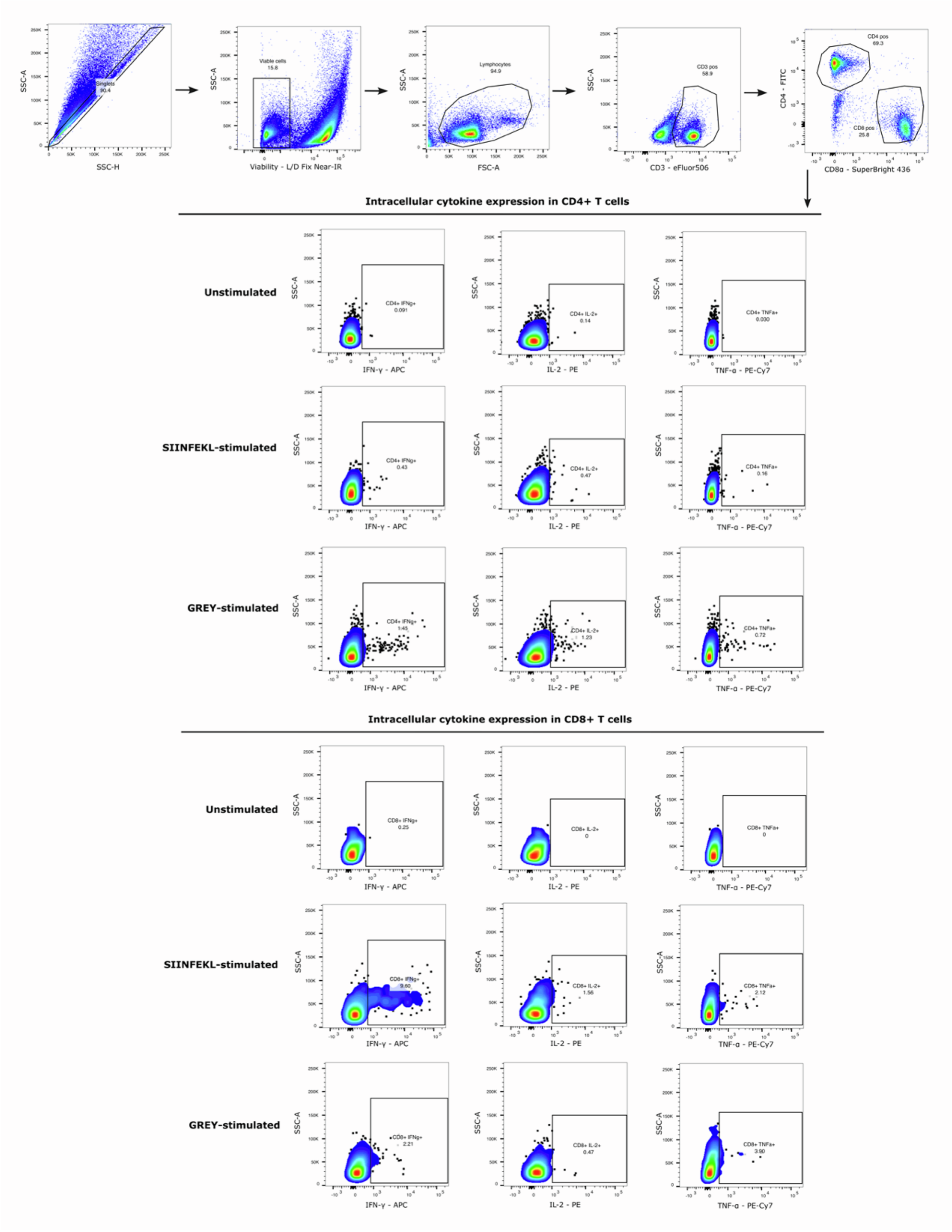

Figure S9. Gating strategy for the specific T cell activation of overnight-peptide stimulated splenocytes. Viable T cells are first gated as Lymphocytes in FSC vs SSC dot plot and then gated as $CD3^+$, following by either $CD4^+$ or $CD8^+$ T cells. Intracellular cytokine staining was performed to analyze peptide-specific $CD4^+$IFN-γ^+, $CD4^+$IL-2^+, $CD4^+$TNF-α^+, $CD8^+$IFN-γ^+, $CD8^+$IL-2^+ and $CD8^+$TNF-α^+ T cells.

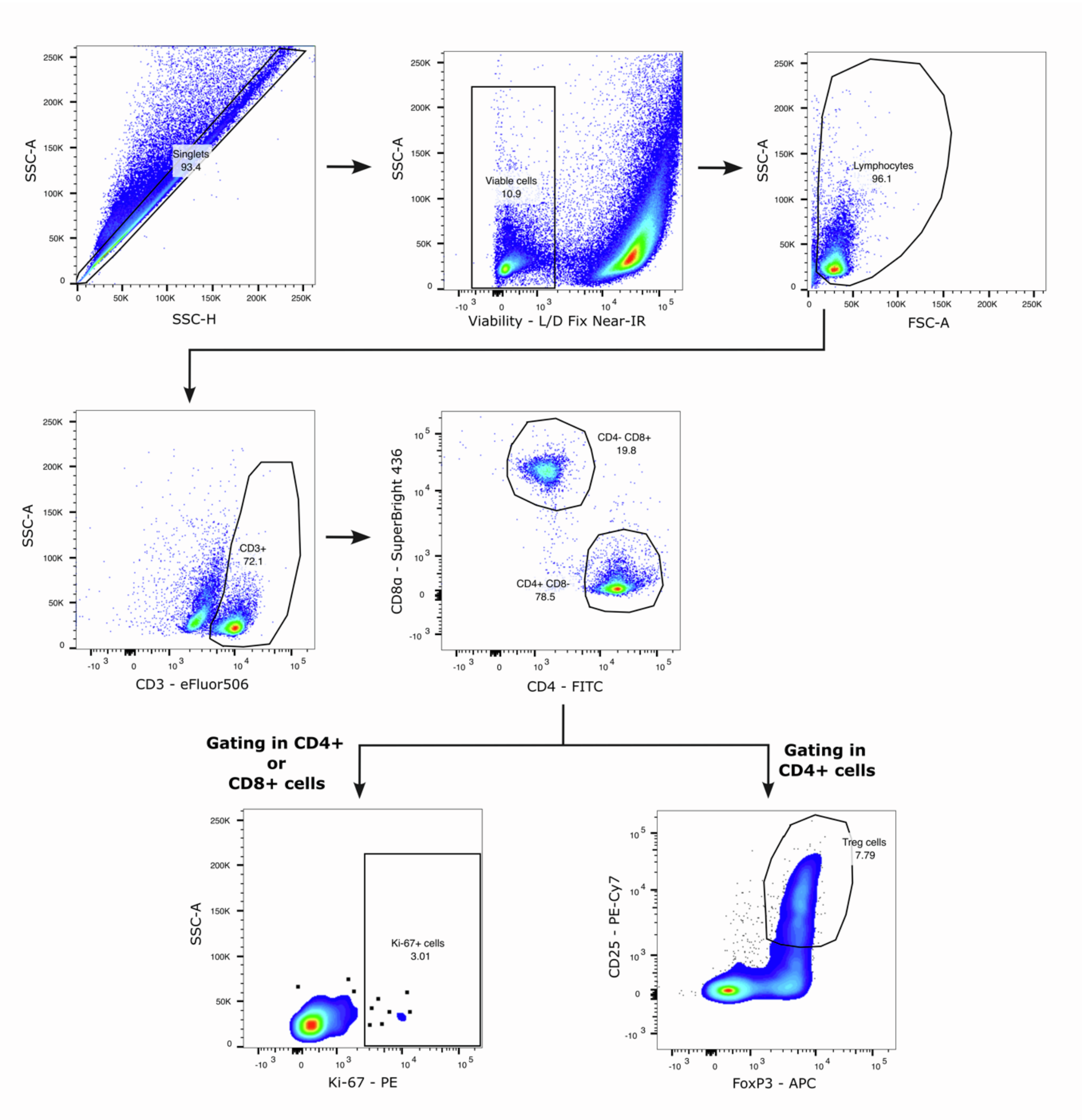

Figure S10. Gating strategy for the specific T cell proliferation and Treg response of 48h-peptide and OVA protein stimulated splenocytes. Viable T cells are first gated as Lymphocytes in FSC vs SSC dot plot and then gated as $CD3^{+}$, following by either $CD4^{+}$ or $CD8^{+}$ T cells. Intranuclear staining was performed to analyze the specific proliferation of $CD4^{+}Ki\text{-}67^{+}$ and $CD8^{+}Ki\text{-}67^{+}$ T cells and the proportion of Treg cells gated as $CD4^{+}CD25^{+}FoxP3^{+}$ T cells.
