# Supplementary material for: mRNA-LNP vaccine strategies: Effects of adjuvants on non-parenchymal liver cells and tolerance
Source: Mol Ther Methods Clin Dev. 2025 Feb 4;33(1):101427. doi: 10.1016/j.omtm.2025.101427 (PMC11872076; doi:10.1016/j.omtm.2025.101427)
Supplement: Document S2. Article plus supplemental information [file mmc2.pdf]

Molecular Therapy
Methods & Clinical Development
Original Article

mRNA-LNP vaccine strategies: Effects of adjuvants on non-parenchymal liver cells and tolerance

Malin Svensson,[1,4] María José Limeres,[1,4] Yanira Zeyn,[2] Rocio C. Gambaro,[1] German A. Islan,[1] Ignacio Rivero Berti,[1] Silvia Fraude-El Ghazi,[1] Leah Pretsch,[1] Katja Hilbert,[1] Paul Schneider,[2] Leonard Kaps,[2,3] Matthias Bros,[2] Stephan Gehring,[1] and Maximiliano L. Cacicedo[1]

[1]Children's Hospital, University Medical Center Mainz of the Johannes Gutenberg University, Langenbeckstr. 1, 55131 Mainz, Germany; [2]Department of Dermatology, University Medical Center of the Johannes Gutenberg University Mainz, Langenbeckstraße 1, 55131 Mainz, Germany; [3]Department of Medicine II Saarland University Medical Center Saarland University 66421 Homburg, Germany

The liver, which plays pivotal roles in metabolism and immunity, often confers tolerance, suppressing immune responses to pathogens. Adjuvanted, lipid nanoparticle-encapsulated mRNA vaccines (mRNA-LNPs) offer a promising approach to overcome immune tolerance. In this study, the immunostimulatory activity of well-documented adjuvants, *i.e.*, 2′3′-cyclic guanosine monophosphate-adenosine monophosphate (cGAMP), resiquimod (R848), and polyinosinic:polycytidylic acid (Poly I:C), on non-parenchymal liver cells was determined. When co-applied with mRNA-loaded LNPs, these adjuvants enhanced immune responses at variable extents. Moreover, the efficiency of mRNA translation in the presence of cGAMP was comparable with the non-adjuvanted control. Repetitive co-application of adjuvants with mRNA-LNPs showed improvement in cellular responses when R848 or R848/cGAMP treatments were used. These findings emphasize the need to delineate the delicate balance between immunomodulatory properties and the efficiency of mRNA translation when selecting adjuvants for mRNA-LNP vaccines and offer insights on how to enhance immunity to infectious diseases and cancers that affect the liver.

INTRODUCTION

The liver filters circulating blood that enters via the hepatic artery and portal vein.[1] Consequently, the liver constantly encounters large quantities of innocuous antigens such as gut-associated bacteria, bacterial products, harmless food-derived antigens, and cellular debris. Immune tolerance to these antigens is fundamental to maintain homeostasis. Tolerance is controlled in large part by various populations of conventional and unconventional antigen presenting non-parenchymal cells (NPCs) consisting of Kupffer cells (KCs), dendritic cells (DCs), and liver sinusoidal endothelial cells (LSECs).[1–3] Notably, the same cell populations play pivotal roles in recognizing antigens as non-self, i.e., danger signals, and eliciting potent *ad hoc* immune responses.

KCs secrete anti-inflammatory, immunosuppressive cytokines (e.g., interleukin [IL]-10, transforming growth factor [TGF]-β) in response to the continued exposure to bacterial endotoxin.[2] Similarly, hepatic DCs, which differ fundamentally from the DCs that reside outside the liver, secrete large amounts of IL-10.[4] The presence of high IL-10 concentrations in the microenvironment contribute to the low-level, cell-surface expression of major histocompatibility complex (MHC I and MHC II) and co-stimulatory molecules, which is characteristic of KCs, DCs, and LSECs residing in the liver under steady-state tolerogenic conditions.[4]

This tolerogenic microenvironment is characterized by T cell dysfunction that occurs due to clonal deletion, anergy, senescence, deviation, exhaustion, and the expansion of the Foxp3^{+} regulatory T (T_{reg}) cell population.[5] The generation of T_{reg} cells depends on factors secreted by NPCs under steady-state conditions.[6] For example, TGF-β, secreted mainly by KCs and all-trans retinoic acid produced by LSECs, are essential for the production and maintenance of T_{reg} cells.[7] Additionally, IL-10 is required to stabilize Foxp3 and to maintain the suppressive T_{reg} cell phenotype.[8] Furthermore, T_{reg} cells inhibit hepatic DC maturation by physical interaction, thereby preventing up-regulated expression of CD80 and CD86 culminating in a tolerogenic DC state.[5,8,9]

Immune tolerance may prevent the liver from recognizing foreign antigens associated with infection or malignancy, resulting in chronic pathologies.[9,10] Novel liver-directed vaccine approaches are urgently needed to overcome the consequences of tolerance and reverse ineffective innate and adaptive immune responses in this organ. The molecular mechanisms involved in regulating tolerance and strategies to boost immunity in the liver remain under investigation.[11–14] In this

Received 15 August 2024; accepted 31 January 2025;
https://doi.org/10.1016/j.omtm.2025.101427.

[4]These authors contributed equally

Correspondence: Maximiliano L. Cacicedo, Children's Hospital, University Medical Center Mainz of the Johannes Gutenberg University, Langenbeckstr. 1, 55131 Mainz, Germany.
E-mail: mcaciced@uni-mainz.de

© 2025 The Author(s).
Published by Elsevier Inc. on behalf of The American Society of Gene and Cell Therapy.
This is an open access article under the CC BY-NC-ND license (http://creativecommons.org/licenses/by-nc-nd/4.0/).

regard, adjuvanted immunotherapies offer a means to stimulate strong immune responses against infectious diseases and malignancies.[9,11,15,16] However, the cellular mechanisms activated in the liver by different adjuvants remain to be elucidated.

Adjuvants, such as 2′3′-cyclic guanosine monophosphate-adenosine monophosphate (cGAMP), resiquimod (R848), and polyinosinic:polycytidylic acid (Poly I:C), elicit innate immune responses by stimulating pattern recognition receptors (PRRs) expressed intracellularly and extracellularly by antigen-presenting cells (APCs).[17,18] Receptor recognition promotes the cell surface expression of major histocompatibility and co-stimulatory (e.g., CD80 and CD86) molecules, and the secretion of proinflammatory cytokines (e.g., IL-6, IL-12, and tumor necrosis factor [TNF]-α). As such, stimulating PRRs with adjuvants represents a rational approach to reversing tolerance and thereby to enhance hepatic immunity.[2,19]

mRNA-based vaccines and therapeutics that emerged during the COVID-19 pandemic promise a novel approach to combat infection, cancer and metabolic diseases.[20,21] In addition to delivering the message encoding the vaccine antigen, mRNA act as a self-adjuvant stimulating toll-like receptors (TLRs), retinoic acid-inducible gene-I (RIG-I), and melanoma differentiation-associated gene 5 (MDA-5), resulting in the release of various cytokines.[22] Such a strong immunological response to mRNA inhibits the translation and synthesis of the encoded protein. However, incorporation of modified nucleosides into the message sequence partially renders the molecule immunosilent.[23] The lipids that comprise the nanoparticles, which form mRNA delivery vesicles, are also capable of stimulating innate immunity although the mechanisms are not yet clear. In general, nucleoside-modified lipid nanoparticle-encapsulated mRNA vaccine (mRNA-LNPs) induce robust humoral and cell-mediated immune responses.[24] Within the liver, however, this may not be enough to circumvent tolerance. Supplementation of mRNA-LNP vaccines with adjuvants offers an approach to overcome this problem.

An ideal adjuvant for mRNA-based vaccines would boost immunity while maintaining optimal translation efficiency.[25] The current study was undertaken to compare the effects of well-known adjuvants, i.e., R848, cGAMP, and Poly I:C, on freshly isolated liver NPC subpopulations. Further, adjuvants in combination with mRNA-LNPs were tested to determine the ability of novel vaccine strategies to overcome the protolerogenic immunophenotype of liver NPCs. The results presented herein demonstrate the capacity of adjuvants to up-regulate co-stimulatory molecule expression and to induce pro-inflammatory cytokine secretion by NPCs without formulation in the LNP structure. Importantly, the data generated highlight the importance of the delicate balance between the immunomodulatory properties of adjuvants and their effects on mRNA translation efficiency and the consequent immune response generated upon its incorporation into an mRNA-LNP vaccine.

RESULTS

Effects of adjuvants on liver NPCs

The abilities of R848, Poly I:C, and cGAMP to activate different liver NPC populations in culture were tested. Activation was evaluated by assessment of the expression of the co-stimulatory markers CD80 and CD86. None of the adjuvants tested up-regulated CD86 expression by $CD45^{+}F4/80^{+}$ KCs; only treatment with R848 upregulated CD80 (Figures 1 and S1). However, R848 treatment up-regulated the expression of both CD86 and CD80 by $CD45^{+}CD11c^{+}$ DCs. Poly I:C and cGAMP promoted slight albeit not statistically significant increases in CD86 expression. Both R848 and cGAMP stimulated marked up-regulation in CD86 expression by $CD45^{+}CD32b^{+}$ LSECs. Only R848, however, induced a significant increase in CD80 in LSECs at every tested dose. Conversely, cGAMP induced activation at the CD80 level only when the dose was significantly increased (Figure S1).

Combined adjuvant effects

NPC populations were treated with a combination of Poly I:C, R848, and/or cGAMP *in vitro* in an effort to stimulate different cell receptors and induce strong innate immune responses. Additive effects were frequently observed when cell populations were treated with a combination of adjuvants compared with treatment with either adjuvant alone. KCs treated with cGAMP combined with either R848 or Poly I:C, for example, exhibited a detectable, albeit statistically insignificant increase in CD86 expression (Figure 2A). In contrast, only treatment with R848 added alone enhanced CD80 expression by KCs.

Non-parenchymal liver DCs exhibited similar trends regarding the expression of CD80 and CD86 in response to adjuvant treatment. Cells treated with a combination of cGAMP and either R848 or Poly I:C exhibited a marked increase in CD86 expression relative to cells treated with cGAMP alone. CD80 expression, in contrast, was only up-regulated by treatment with R848 alone. In the case of LSECs, treatment with single adjuvants induced modest increases in CD80 and CD86 level, whereas adjuvant combinations induced substantially increased levels.

The response to adjuvants added alone or in combination was studied further by quantifying cytokine and chemokine production by cells stimulated in culture. Although varied in extent, R848 added alone to NPC cultures increased the production of most cytokines and chemokines tested (Figure 2B). In comparison, cGAMP added alone induced comparatively less production of most cytokines (aside from interferon [IFN]-α and IFN-β) though production was greater than that determined for untreated, control cultures. Similarly, IP-10, RANTES, and MCP-1 concentrations were greater in cGAMP-treated NPC cultures, relative to the untreated control group. Likewise, cytokine/chemokine concentrations were often elevated in NPC cultures treated with Poly I:C alone though generally less than those determined in cultures treated with either R848 or cGAMP.

NPCs cultured with R848 combined with Poly I:C or cGAMP exhibited modest increases in cytokine production compared with cells

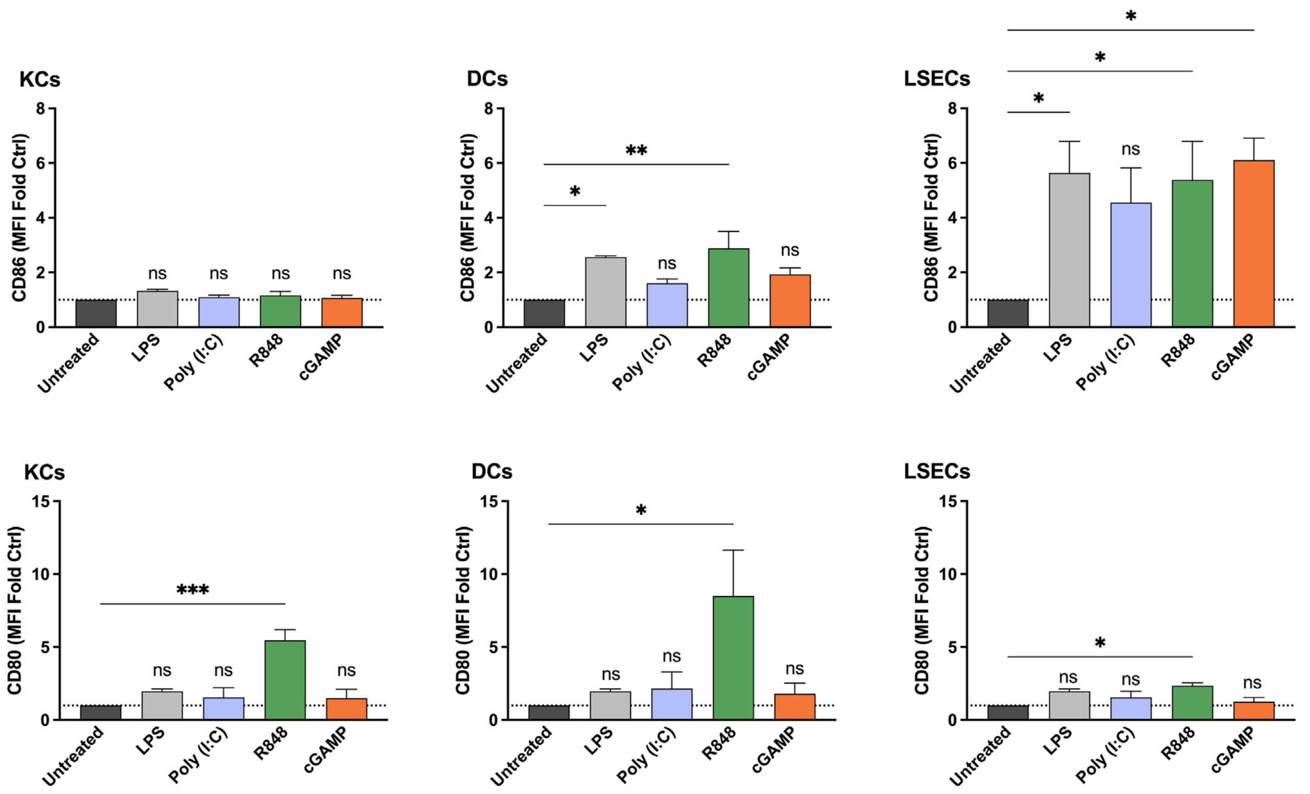

Figure 1. Adjuvants stimulate the expression of CD86 and CD80 by NPC subpopulations

NPC cultures were treated overnight with 5 μg/mL R848, Poly I:C, or cGAMP; cells treated with 1 μg/mL LPS served as a positive control. CD86 and CD80 expression was evaluated by flow cytometry in KCs, DCs and LSECs. Data are the means ± SEM (n = 3). Significantly different from the control (untreated): ns, not significant; *$p < 0.05$; **$p < 0.01$ (one-way ANOVA, Dunnett's multiple comparison test). MFI, mean fluorescent intensity.

cultured with the adjuvant alone. The levels of IFN-γ, TNF-α, and IL-6 produced in cultures treated with R848 and Poly I:C, for example, were 50%–60% greater than those produced in cultures treated with R848 only; the concentrations of IFN-β and IFN-α were nearly 2-fold and 5-fold greater, respectively. Similarly, modest increases in the levels of IFN-γ, TNF-α, IFN-α, and IFN-β were determined in cultures treated with a combination of R848 and cGAMP relative to cultures treated with either adjuvant alone; RANTES, IP-10, and MCP-1 concentrations were also increased. cGAMP combined with Poly I:C stimulated slight increases in IFN-α, IFN-β, IL-6, TNF-α, RANTES, and MCP-1 relative to NPCs cultured in the presence of either adjuvant separately.

Adjuvant-stimulated T cell proliferation

To evaluate the effects of R848, cGAMP, and Poly I:C on the capacity of liver NPCs to stimulate T cell proliferation, ovalbumin (OVA)-responsive $CD8^+$ and $CD4^+$ T cells were co-cultured with OVA protein- or peptide-pulsed NPCs (Figure 3A). Cell proliferation rates were evaluated and compared upon addition of adjuvants alone or in combination. It is noteworthy that OVA-stimulated liver NPC cultures exhibited higher $CD8^+$ T cell proliferation when pretreated with R848 plus cGAMP compared with cultures treated with a combination of R848 and Poly I:C (Figure 3B). However, none of the adjuvant combinations conferred stronger $CD8^+$ T cell proliferation by SIINFEKL-pulsed NPC than pretreatment with individual adjuvants. A significant increase in the proliferation of OVA-responsive $CD4^+$ T cells was also found in OVA protein-pulsed NPC cultures treated with both R848 and cGAMP. Similarly, the combinations of R848 with either cGAMP or Poly I:C stimulated the proliferation of $CD4^+$ T cells co-cultured with the GREY-pulsed NPCs; however, proliferation was not significantly greater than that determined in co-cultures treated with R848 alone.

The secretion of cytokines and chemokines was increased by the addition of R848 and cGAMP or R848 and Poly I:C to co-cultures composed of purified, antigen-specific $CD8^+$ and $CD4^+$ T cells and OVA- or peptide-pulsed NPCs (Figure 3C). For the most part, cytokine/chemokine release in co-cultures treated with adjuvants containing antigen-specific $CD8^+$ T cells and peptide- or OVA-pulsed NPCs was greater than that observed in antigen-specific $CD4^+$ T cell co-cultures.

Mouse non-parenchymal liver cells respond to *OVA* mRNA-LNPs administered with adjuvants

As an initial approach to determine the impact of adjuvants on the response to mRNA-based vaccines, NPC activation was studied in

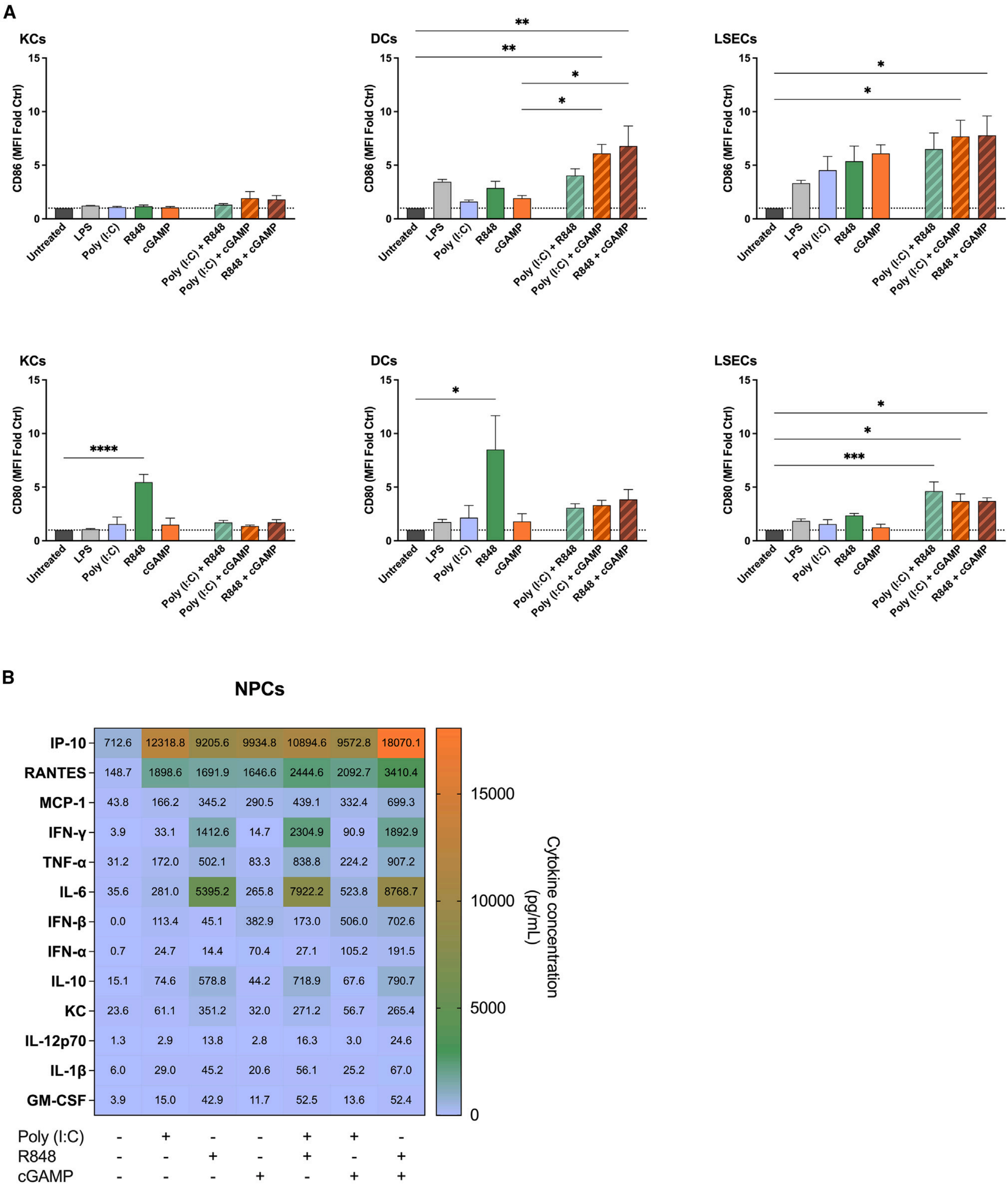

(legend on next page)

mice injected intravenously (i.v.) with nucleoside-modified *OVA*-encoding mRNA formulated into LNPs co-applying R848, cGAMP or a combination of both (Figure 4A). *OVA* mRNA-LNPs alone (LNPs-OVA) failed to upregulate the expression of either CD86 or CD80 co-stimulatory molecules by KCs, DCs, or LSECs when assessed 24 h after single injection (Figure 4B). LNPs-OVA administered with cGAMP alone or in combination with R848 up-regulated the expression CD80 or CD86 by KCs, DCs, and LSECs compared with NPCs derived from mice that have been administered cGAMP alone. However, cGAMP did not partition into the LNP structure (Figure S2). Meanwhile, R848 increased activation markers to a similar extent either when administered alone or with LNPs-OVA.

Additionally, NPCs derived from mice vaccinated with LNPs-OVA with or without adjuvants were cultured. The supernatants were collected after 24 h of incubation, and cytokine/chemokine production was quantified by cytometric bead array (CBA). The administration of LNPs-OVA alone had a negligible effect on cytokine/chemokine secretion. Cytokine/chemokine release by NPCs derived from control (PBS) and LNPs-OVA-treated mice was comparable (Figure 4C). LNPs-OVA administered in combination with adjuvants, however, promoted cytokine/chemokine secretion. The according concentrations were particularly elevated in supernatants collected from cultures of NPCs derived from mice vaccinated with LNPs-OVA and a combination of cGAMP and R848. Notably, the increase in IFN-α concentration was marginal.

R848 impairs LNP-encapsulated mRNA translation

To determine the adjuvant effects on mRNA translation, the former were mixed rapidly with *Luc* mRNA-LNPs and administered i.v. into mice. Luciferase activity was measured *in vivo* and *ex vivo* at 6 h after treatment (Figure 5A). *Luc* mRNA-LNPs (LNPs-Luc) control mice expressed a strong signal localized mainly in the liver (Figure 5B). LNPs-Luc administered in combination with cGAMP also showed a marked signal around the liver, though no fluorescence was detected in 2 animals. The cohorts that had been administered LNPs-Luc with R848 or with R848 and cGAMP exhibited marked decreases in Luc expression as compared with the control group. Immediately after tissue collection, the luciferase signal was measured *ex vivo* as shown in Figure 5C. Quantitation of the fluorescence detected in the liver determined comparable values in mice administered LNPs-Luc and those administered LNPs-Luc with cGAMP. However, fluorescence was significantly diminished in the livers of mice inoculated with LNPs-Luc and R848 with or without cGAMP. In this context, no interaction between cGAMP and the LNPs could be detected (Figure S1).

Repetitive administration of *Ova* mRNA-LNP in combination with adjuvants led to distinct immune responses

Experimental design and safety

Based on the obtained results on cell-specific activation and antigen expression, an immunization study using LNPs-OVA with adjuvants was performed in order to assess how these formulations modulate antigen-specific immunity. Mice were injected i.v. three times, 5 days apart, as depicted in Figure 6A. The experimental endpoint occurred on day 11, where the liver and spleen tissues were collected for cell isolation and study. Of note, treatment-induced liver toxicity was evaluated by the activity of liver transaminases (alanine aminotransferase and aspartate aminotransferase) after treatment and appearance of the extracted organs. No acute toxic effects were observed in any of the treated animals, reflected by the absence of abnormal behavior and no significant differences in bodyweight compared with a PBS control (Figures S3 and S4).

Immune cell activation

In contrast with the single-injection experiment, no significant up-regulation of activation markers on liver NPCs was observed after repetitive injections compared with LNPs-OVA alone (Figure 6B). Moreover, mRNA-LNP without adding adjuvants increased the frequencies of $CD86^+$ cells for LSEC, DC, and KC populations. In the case of liver DCs, LNPs-OVA significantly up-regulated the levels of the MHC II marker (Figure 6B). Previous observations showed an absence in cell activation to LNPs-OVA after a single injection (Figure 4B). Notably, the co-application of R848 with LNPs-OVA arrested the increment in activation markers observed by LNPs-OVA alone.

However, splenocytes showed a higher response to adjuvant treatments. For DC or macrophage, R848 significantly increased the frequencies of $CD80^+$, $CD86^+$, and MHC II^+ cells (Figure 6C). Concurrently, cGAMP showed stimulation on DCs and macrophages at CD86 level. Moreover, the expression of CD80 was increased by cGAMP treatment in macrophages. Co-application of R848/cGAMP resulted in no significant differences in comparison to R848 treatment alone (Figure 6C).

Antigen-specific immune responses

Antigen-specific cell-mediated immune responses were characterized by evaluating levels of cytokine-secreting $CD4^+$ and $CD8^+$ T cells (Figure 6D). After stimulation using MHCI-restricted (SIINFEKL) or MHCII-restricted (GRAY) peptides, T cells secreting IFN-γ, IL-2, or TNF-α were identified. Co-administration with R848/cGAMP led to the highest levels of cytokine-secreting $CD4^+$ T cells when stimulated with SIINFEKL. In contrast, when T cells were incubated with GREY, no additive effect was observed by adjuvants in

Figure 2. Individual or a combination of adjuvants stimulates the expression of CD86 and CD80 by NPCs

NPC cultures were treated overnight with 5 μg/mL of each adjuvant; cells treated with 1 μg/mL LPS served as positive controls. CD86 and CD80 were evaluated by flow cytometry in KCs, DCs and LSECs. Data are the means ± SEM (n = 3).

Significantly different between groups: *$p < 0.05$; **$p < 0.01$; ***$p < 0.001$; ****$p < 0.0001$ (one-way ANOVA, Tukey's multiple comparison test) (A). Cytokines in the supernates of NPCs culture treated with adjuvants were quantified by CBA (B). MFI, mean fluorescent intensity.

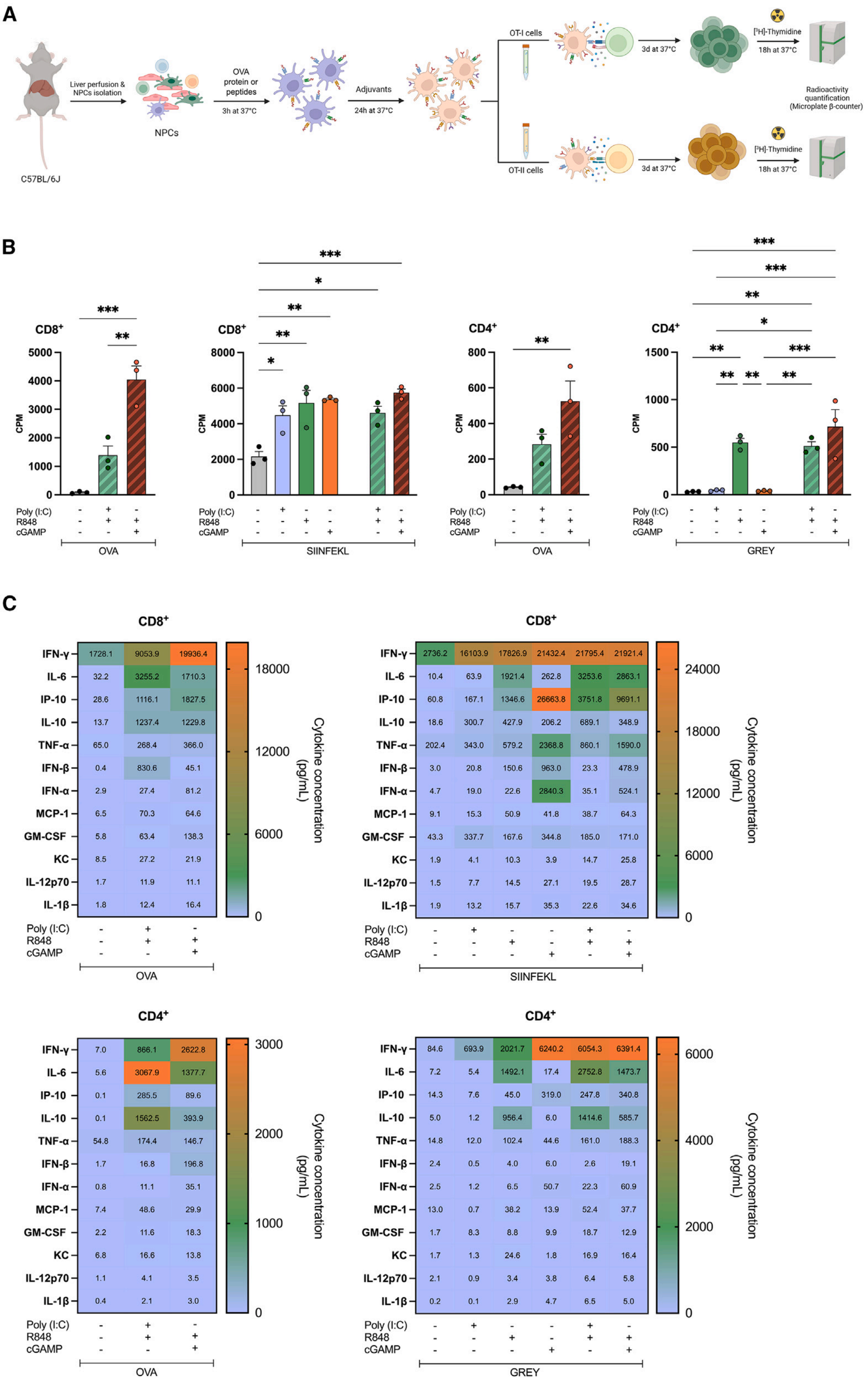

(legend on next page)

comparison with the positive effect generated by LNPs-OVA alone (Figure 6D). Cytokine secretion by $CD8^+$ T cells was stronger when R848 was administered along with LNPs-OVA. These effects were observed only with R848 co-applied alone with LNPs-OVA. Cytokine levels collected from culture supernates paralleled this observation (Figure S5).

In addition, antigen-specific T cell proliferation was evaluated. Both $CD4^+$ and $CD8^+$ T cells displayed elevated proliferation rates when immunized with LNPs-OVA administered with R848 alone (Figure 6E). Noteworthy, R848/cGAMP also led to increased proliferation but only in $CD4^+$ T cells.

Humoral immune response

A pertinent finding was observed regarding the production of anti-OVA antibodies. All treated groups displayed significant antibody titers (Figure 6F). However, no differences were observed between the groups.

T_{reg} cell response

Elevated T_{reg} levels are associated with the failure of anti-cancer immunotherapies.[26] Here, the percentage of T_{reg} cells after co-application of adjuvant and LNPs-OVA was assessed by the frequency of $CD4^+$ $CD25^+$ $FoxP3^+$ T cells. Treatment with LNPs-OVA alone led to a significant increase in T_{reg} cells at the experimental endpoint. Treatment with cGAMP exerted similar effects. Conversely, single or combine treatment with R848 did not show a significant increase in T_{reg} cells compared with the untreated control group (Figure 6G).

DISCUSSION

The fragile balance between immunity and tolerance in the liver is mediated, in part, by the NPCs, i.e., KCs, DCs, and LSECs.[1,2] Reversing tolerance to induce therapeutic responses to persistent viral infections and cancers is a frequent topic of discussion. An improved understanding of the response of NPCs to adjuvants would help to optimize the design of liver-directed therapies that include mRNA-based technologies. Three well-known adjuvants, cGAMP, R848, and Poly I:C, were selected for the current study based on their immunologic potencies and distinct structural characteristics and mechanisms of action.

cGAMP is a cyclic dinucleotide that participates in the oligomerization and subsequent activation of the stimulator of IFN genes protein. It promotes cancer immunity by inducing type I IFN production, including the expansion of antigen-specific $CD8^+$ T cells.[27,28] R848 is an imidazoquinoline that activates TLR7/8 and thus stimulates DC maturation, up-regulates co-stimulatory molecule expression, and promotes pro-inflammatory cytokine secretion.[29] R848 increases the number of infiltrating $CD8^+$ T cells and decreases T_{reg} cell frequencies when used in cancer treatment.[30] Poly I:C is a double-stranded RNA molecule that triggers TLR3, MDA-5, and RIG-I receptors resulting in type I IFNs production, Th1-polarizing cytokine secretion and overall improved T cell responses.[28]

In the current study, liver NPCs were treated with cGAMP, R848, and Poly I:C in parallel settings, and the responses were assessed and compared. R848 treatment up-regulated CD86 and/or CD80 expression by all three monitored NPC populations: KCs, DCs, and LSECs. KCs and liver DCs, however, were unresponsive toward other adjuvants. Furthermore, treatment with either cGAMP or Poly I:C, as well as R848, up-regulated the expression of the cell-surface costimulatory molecules by LSECs. Notably, LSECs display many characteristics typical of APCs and exhibit the capacity to cross-present antigens.[6] Indeed, LSEC stimulation was demonstrated to improve T cell-mediated immunity in mice.[31]

Remarkably, R848 combined with cGAMP or Poly I:C synergized, up-regulating stronger CD80 and CD86 expression by liver DCs and LSECs, but not KCs, than treatment with any single adjuvant. As expected, combining adjuvants that exert their effects by different mechanisms maximized immunostimulatory potency. The adjuvant systems developed by GlaxoSmithKline constitute a rational combination of classical adjuvants like alum with novel immunostimulators like TLR agonists. This proved to be a potent and reliable approach to optimizing immune responses.[32]

Cheng et al.[33] explored adjuvant combinations in a nanoformulation. R848 and cGAMP combined with immune checkpoint inhibitors reduced immunosuppression in a tumor microenvironment, and elicited anti-tumor immunity in a number of mouse models.[33] Reportedly, the combination of R848 and Poly I:C also offers a successful strategy to activating DC subsets circulating in the blood. In this regard, Hänel et al.[34] found that activating human DCs in the circulation stimulated natural killer cells and potent antigen-specific T cell immunity. Similarly, Anfray et al.[35] reported that in a mouse lung cancer model treatment with a nano-formulated combination of R848 and Poly I:C reprogrammed tumor-associated macrophages by increasing the M1 ($F4/80^+$,$CD86^+$) by M2 ($F4/80^+$,$Arg1^+$) ratio and suppressing tumor progression. To the best of our knowledge, the effects of adjuvant combinations on liver NPC have not been studied to date.

Figure 3. NPCs pre-treated with R848, cGAMP and Poly I:C exert a distinctive capability to stimulate T cell proliferation

Experimental scheme. NPCs were isolated from C57BL/6JRj mice, pulsed with OVA or peptides (SIINFEKL or GRAY), and stimulated with adjuvants. Then, the NPCs were cocultured with SIINFEKL-specific $CD8^+$ T cells (OT-I) or GREY-specific $CD4^+$ T cells (OT-II); proliferation was quantified by the incorporation of H^3-thymidine (A). Proliferation of OVA-specific $CD4^+$ and $CD8^+$ T cells cocultured with OVA- or peptide-pulsed NPCs in the presence of individual or combined adjuvants. Data are the means ± SEM ($n = 3$). Significantly different between groups: *$p < 0.05$; **$p < 0.01$; ***$p < 0.001$; (one-way ANOVA, Tukey's multiple comparison test). (B). Cytokine secretion in cocultures comprised of OVA-specific $CD4^+$ or $CD8^+$ T cells, and NPCs pulsed with OVA, SIINFELK, or GREY in the presence of individual or combined adjuvants (C). Data are the means ± SEM ($n = 3$). Significantly different from the control: ns, not significant; *$p < 0.05$; **$p < 0.01$; ***$p < 0.001$; ****$p < 0.0001$ (one-way ANOVA).

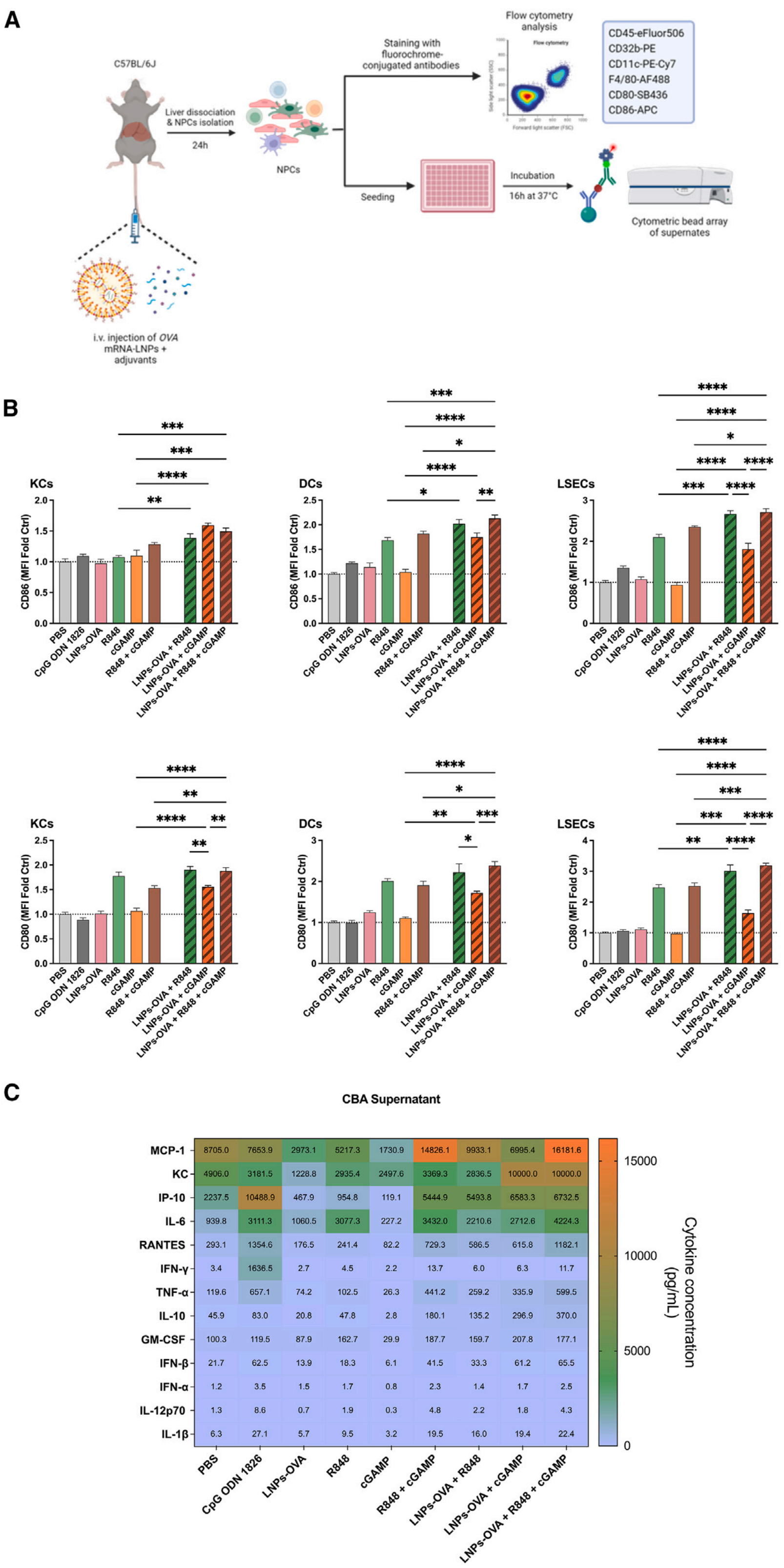

Figure 4. Adjuvants co-administered with mRNA-LNPs lead to the up-regulation of co-stimulatory markers in liver NPCs, after single i.v. injection in mice

Experimental scheme. C57BL/6J mice were injected i.v. with LNPs-OVA and adjuvants. The NPCs were isolated and separated into two fractions. The NPC activation in one fraction was quantified by flow cytometric analysis. The NPCs in the second fraction were cultured; the supernates were collected and the cytokine/chemokine profile was evaluated (A). CD86 and CD80 expression by NPC subpopulations; i.e., KCs, DCs, and LSECs; isolated from mice inoculated with OVA mRNA-LNPs and adjuvants alone or in combination was assessed. Data are the means ± SEM (n = 5). Significantly different between groups: *$p < 0.05$; **$p < 0.01$; ***$p < 0.001$; ****$p < 0.0001$ (one-way ANOVA, Tukey's multiple comparison test) (B). Cytokines and chemokines in the NPCs culture supernates were quantified by CBA (C).

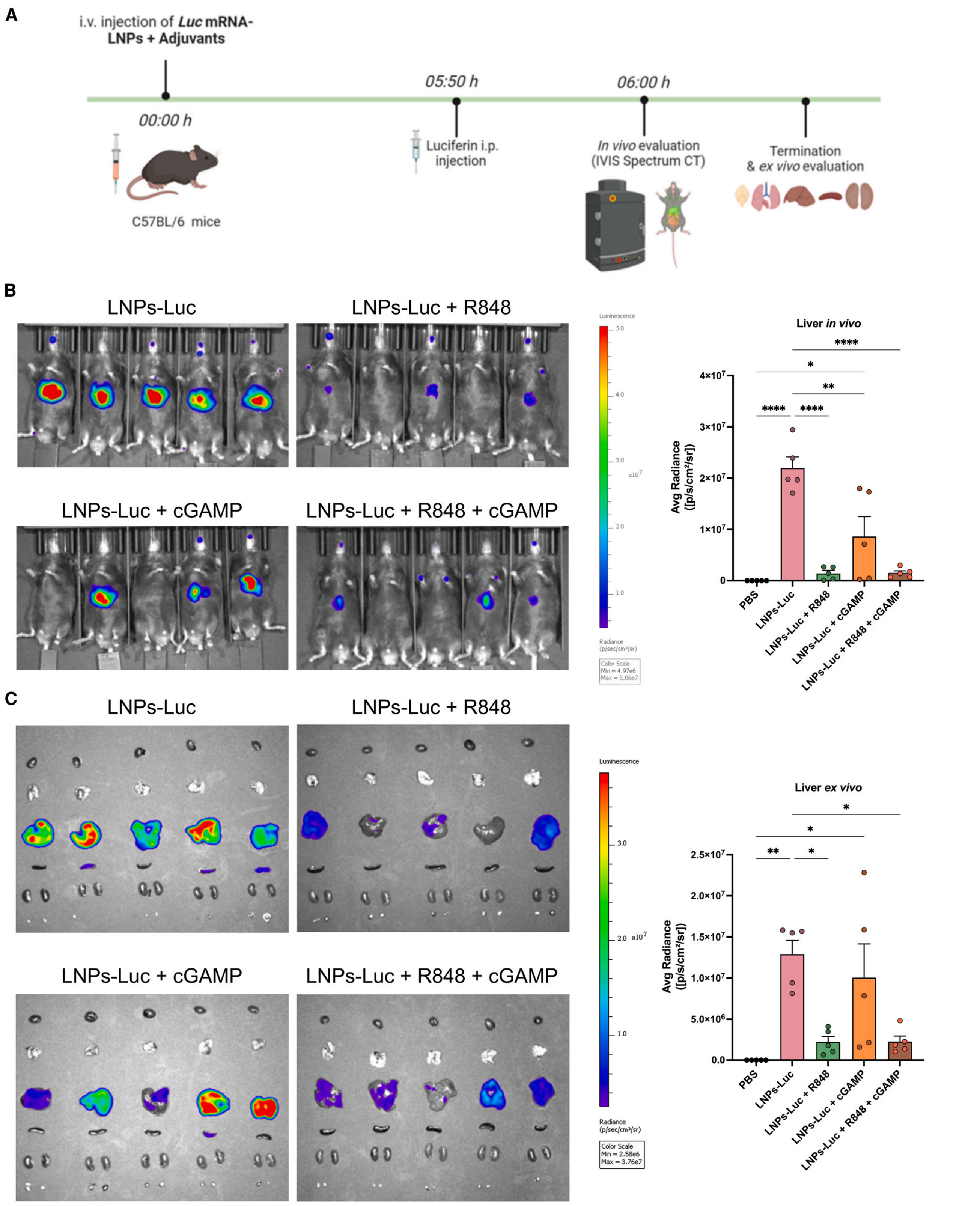

(legend on next page)

To clarify the adjuvant effect of these agents on the liver, NPC loaded with OVA-derived antigen were cocultured with OVA antigen-specific $CD8^+$ and $CD4^+$ T cells. Cocultures composed of liver NPCs pre-treated with R848, cGAMP, or Poly I:C alone or in combination and T cells stimulated comparable increases in $CD8^+$ T cell proliferation. In the case of $CD4^+$ T cells, however, increased proliferation occurred only in cocultures containing liver NPCs pre-treated with R848 and either cGAMP or Poly I:C.

The use of synthetic mRNA-LNP formulations in therapeutic approaches to prevent or treat diseases has strongly expanded over the past years. The LNPs in these formulations perform two primary functions at the same time: transport mRNA molecules inside of cells and provide adjuvant effects.[20] These effects are not well understood from a mechanistic point of view.[24] The co-application of well-known adjuvants into mRNA-LNP formulations provides a rational approach to predict the effects of adjuvants *in vivo* and to potentiate innate immunity. The latter is extremely relevant in situations where reversing immune tolerance is paramount, e.g., cases of liver pathologies. While various adjuvants have been shown to stimulate liver NPCs, none have been co-applied with mRNA-LNPs to treat liver diseases.[21,36]

In the current study, LNP-encapsulated *OVA* mRNA combined with cGAMP and R848 were injected into mice to determine the effects of these adjuvants on liver NPC activity. Liver tissues were targeted rapidly by simply mixing adjuvants with mRNA-LNPs and injecting i.v. Similar to the results obtained *in vitro*, adjuvants administered alone failed to activate KCs. KCs were activated, however, when either R848 or cGAMP was administered in combination with LNPs-OVA, indicative of a co-activator property of the transfection complexes. R848 inoculated alone stimulated both DCs and LSECs; stimulation increased perceivably when R848 was administered in combination with mRNA-LNPs. cGAMP, in contrast, only exerted a strong stimulatory effect on either NPC population monitored when co-applied with LNPs-OVA. Notably, LNPs-OVA administered alone as a control never activated NPCs.

Upon systemic administration, blood proteins are adsorbed onto the surface of LNPs forming a corona.[37] As such, it is a matter of conjecture whether the observations reported herein result from the interaction of adjuvants with LNPs or with the protein corona. Other investigators report that the encapsulation of cGAMP in LNPs improved its stability and immunomodulating effects.[38–40] The simple combination of cGAMP with mRNA-LNPs in the study reported here was sufficient to promote KCs, DCs, and LSECs activation.

A delicate balance exists between adjuvant-activated innate immunity and the effects of adjuvants on mRNA translation. Indeed, activation of the innate immune response may impair mRNA translation.[20,41] Here, the effects of cGAMP and R848 on mRNA translation *in vivo* were tested. Our data indicate that R848 exerts a strong negative impact on mRNA translation; cGAMP affected translation to a far lesser extent. A number of previous studies reported improved immune responses to mRNA-LNP formulations that incorporated either R848 or cGAMP; the effects of these adjuvants on mRNA translation, however, were not considered.[25,42,43]

To further investigate the effects of these adjuvants in mRNA-based immunization, a repetitive administration scheme was designed to mimic strategies frequently used in cancer immunotherapy.[44,45] Interestingly, the state of activation of liver DCs, LSECs, and KCs was not affected by the presence of adjuvants. However, the administration of LNPs-OVA elicited up-regulation of the measured activation markers. This observation was counterintuitive, considering the results obtained after single injection, where mRNA-LNP did not show any stimulatory effect. Conceivably, the repetitive administration induced adjuvant-like effects by the LNPs on liver NPCs. It remains a matter of conjecture whether LNPs can exert an effect strong enough to overcome liver tolerance. Further investigation would need to be conducted to understand these events. In contrast, DCs and macrophages isolated from the spleen responded mainly to R848 co-treatment. These findings provided evidence regarding the complexity in the induction of immune activated state by liver NPCs through adjuvant treatments. Notably, cell-mediated immunity was improved by R848 and, to a lesser extent, by the R848/cGAMP combination, evidenced by an increase in the number of cytokine-secreting $CD4^+$ and $CD8^+$ T cells. A similar pattern was observed when the capacity of T cells to proliferate after antigen stimulus was evaluated.

Intriguingly, the production of anti-OVA antibodies did not seem to be affected by the co-application of the adjuvants used in this study. Due to the observed negative impact on mRNA translation by R848, a decrease in total IgG titers was expected. Conceivably, the level of antigenic protein obtained in R848-adjuvanted LNPs-OVA was enough to mount a humoral response similar to the LNPs-OVA alone. Moreover, the boosting effects of the repetitive administrations might have additionally contributed to reaching similar systemic antibody levels between groups. The connection between antigen dose and antibody production responds to a complex relationship and requires further investigations.[46,47]

T_{reg} cells play a crucial role in homeostasis and the induction of tolerance. However, the abundant presence of T_{reg} cells in the tumor

Figure 5. R848 and cGAMP negatively impact mRNA translation after mRNA-LNP i.v. injection in mice
Biodistribution of LNPs-Luc with or without adjuvants was performed as shown in the experimental scheme. Six hours after injection of the formulations, luciferase-associated bioluminescence was measured (A). *In vivo* images were collected in all mice cohorts and luminescence in the region of the liver was quantified (B). The mice were then dissected, and the hearts, lungs, livers, spleens, kidneys, and inguinal lymph nodes were collected for *ex vivo* imaging. Luminescence localized in the liver *ex vivo* was quantified (C). Data are the means ± SEM ($n = 5$). Significantly different between groups: *$p < 0.05$; **$p < 0.01$; ***$p < 0.001$; ****$p < 0.0001$ (one-way ANOVA, Tukey's multiple comparison test).

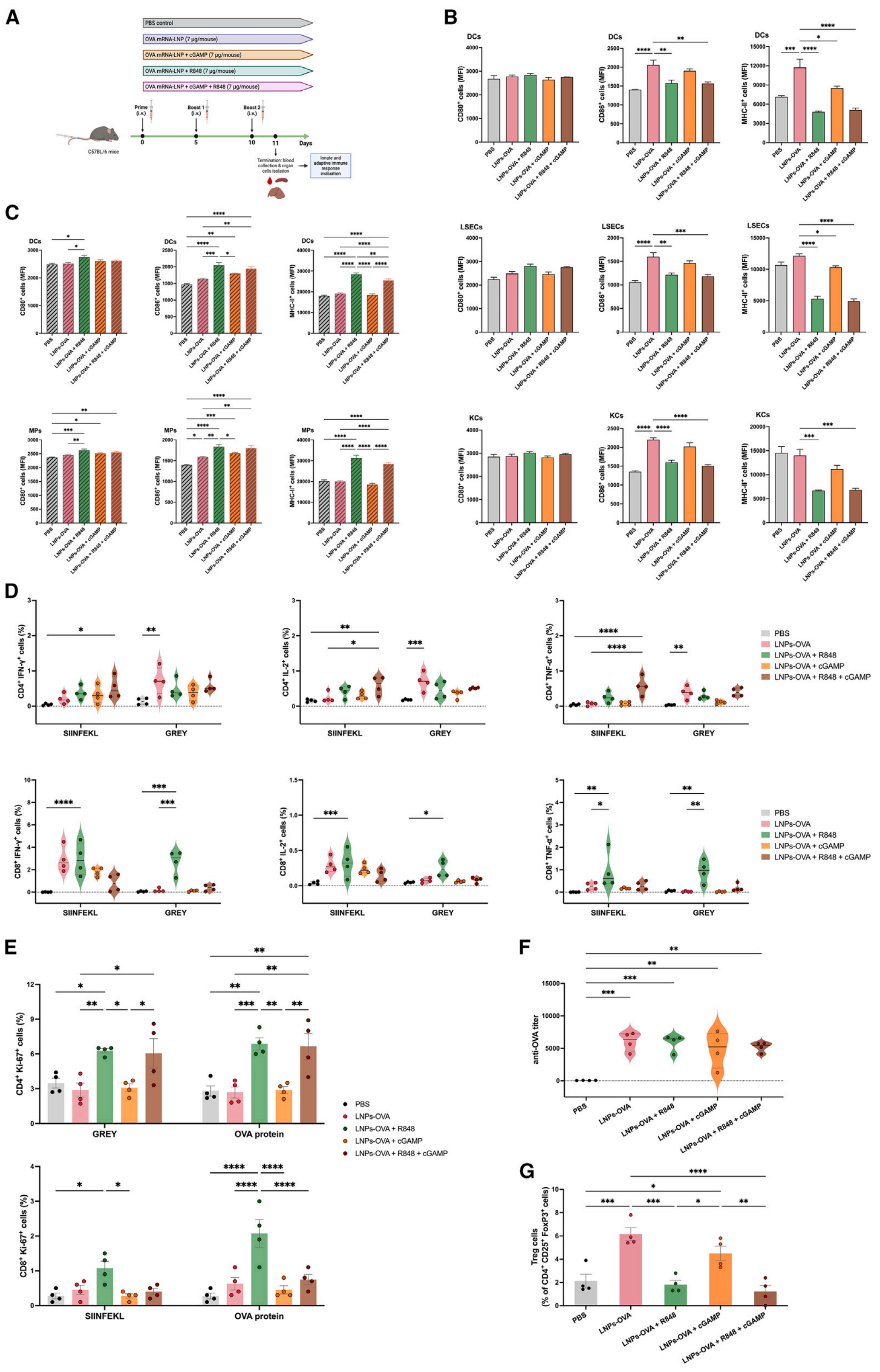

(legend on next page)

microenvironment leads to huge challenges in cancer therapies.[48] Modulation of T_{reg} frequencies, e.g., with adjuvants, is a promising approach to overcome immune tolerance. Remarkably, R848 and R848/cGAMP co-treatments did not induce an increase in the numbers of T_{reg} cells, while mRNA-LNP without adjuvantation showed an effect. Recently, Zhou et al.[49] reported that continuous R848 treatment led to a decrease in the number of T_{reg} cells in tumor-bearing mice. Primarily, the CTL:T_{reg} ratio is often associated with the therapeutic index of a given anti-tumor treatment.[50] The expansion of $CD8^+$ T cells together with a low number of T_{reg} cells indicate a potentially strong anti-tumor effect. Nevertheless, more in-depth studies using R848-adjuvanted mRNA-LNP will need to be carried out to confirm this observation.

The inoculation of mice with mRNA-based vaccines induces production of type I IFNs, which function as a double-edged sword, stimulating T and B cell responses on the one hand, while inhibiting mRNA translation as an anti-viral effector mechanism on the other.[23,25,41,51,52] The results of the present study suggest that an informed selection of adjuvants with an equilibrated impact on mRNA translation may in parallel promote APC activation and the production of antigenic proteins. This is particularly relevant for immunotherapies that target liver tissue, e.g., hepatocellular carcinoma, to overcome intrinsic liver- as well as tumor-induced tolerance and to elicit antigen-specific effector $CD4^+$ and $CD8^+$ T cells collectively contribute to successful therapies.

Conclusions

The intricate balance between immunity and self-tolerance in the liver is governed, in part, by NPCs. Approaches to reverse liver tolerance for therapies to treat viral infections or cancer remain a matter of debate. Understanding the response of NPCs to adjuvants should serve to optimize liver-directed therapies. Adjuvant combinations, especially R848 in combination with cGAMP or Poly I:C, synergized to enhance immune responses. Despite these findings, all adjuvant combinations failed to activate KCs. Co-application of adjuvants with mRNA-LNP formulations potentiated innate immune activation. Notably, a single injection of cGAMP and R848 co-administered with LNPs-OVA activated NPCs and promoted OVA peptide-specific $CD8^+$ and $CD4^+$ T cell proliferation. R848 impacted mRNA translation negatively, however, emphasizing the requirement to balance immune stimulation and translation efficiency. Repetitive injections of adjuvanted LNPs-OVA led to distinct immune responses with a rather poor activation of NPCs by adjuvants. Cellular responses in splenocytes were significantly enhanced by the co-administration of R848 or R848/cGAMP together with the mRNA-LNP formulation. In contrast with the results obtained from LNPs-OVA alone, these adjuvanted treatments did not provoke an increase in tolerogenic T_{reg} cells.

Furthermore, the reduced mRNA translation by R848 seems to be negligible to the immunity generated after a repetitive injection scheme. In summary, the selection of adjuvants for co-administration with mRNA-LNPs relies on the capacity to exert minimal impact on mRNA translation while preserving stimulatory properties. Such a strategy may enhance liver-directed immunotherapies like those designed to treat hepatocellular carcinoma or chronic liver infections, e.g., hepatitis B.

MATERIALS AND METHODS

Mice

Wildtype male C57BL/6JRj mice were purchased from Janvier (Le genest-Saint-Isle, France) and Charles River Laboratories (Wilmington, MA, USA) were maintained in the Central Animal Facilities of the University Medical Center of Mainz under pathogen-free conditions. Five mice/cage were housed in a dedicated vivarium (18°C–23°C ambient temperature, 40%–60% humidity, 14 h:10 h light cycle) with clean food, water, and bedding. Similarly, male and female transgenic OT-I and OT-II mice on a C57BL/6 background were bred and maintained in the Central Animal Facilities. All animal procedures were approved by the local authorities (Landesuntersuchungsamt Rhineland-Palatinate). Ethical approval was granted by the Landesuntersuchungsamt LUA, Koblenz, AK G20-1-123 and G20-1-130.

LNP-encapsulated mRNA

Ova-mRNA and *luciferase*-mRNA were synthesized by TriLink Biotechnologies (San Diego, CA, USA). The mRNA was encapsulated into LNPs using a NanoAssemblr Ignite machine for formulation (Precision Nanosystems, Vancouver, Canada). All procedures were conducted according to the manufacturer's protocols. Briefly, Genvoy-ILM lipid mix (Precision Nanosystems), containing 50% ionizable lipid, 10% DSPC, 37.5% cholesterol, and 2.5% PEG-lipid was mixed with mRNA in 50 mM citrate buffer, pH 4.0, using an N/P ratio of 4. The aqueous and organic phases were mixed at a total flow rate of 12 mL/min and flow rate ratio of 3:1 (aqueous:organic). After synthesis, LNPs were 1:20 diluted or 1:40 in PBS for *in vitro*

Figure 6. Immunization using repetitive LNPs-OVA i.v. injections provide insights in the improvement of cell-mediated immune responses by co-administration with adjuvants

Experimental scheme. Mice were i.v. injected, three times, 5 days apart either with LNPs-OVA alone, with R848 or cGAMP and with both adjuvants combined. The following day after the second booster, whole blood was collected, and spleen and liver were dissected for further analysis (A). Activation of NPCs (B) and splenocytes (C) subpopulations was measure by flow cytometry through up-regulation of the activation markers CD80, CD86 and MHC II. MP, macrophages. Peptide-specific $CD4^+$ and $CD8^+$ T cell responses were measure by the release of intracellular cytokines: IFN-γ, IL-2, and TNF-α. Cells percentages were normalized to their respective $CD3^+$ T cell population (D). T cell proliferation of the $CD4^+$ and $CD8^+$ T cells after 48-h peptide- or OVA-stimulated splenocytes measured by the up-regulation of the intranuclear protein Ki-67. Cells percentages were normalized to their respective $CD3^+$ T cell population (E). Total anti-OVA IgG titers at termination (day 11) of the immunization study (F). T_{reg} response after 48-h GREY-stimulated splenocytes. T_{reg} cells are gated as $CD4^+CD25^+FoxP3^+$ cells (G). Data are the means ± SEM (n = 4). Significantly different between groups: *$p < 0.05$; **$p < 0.01$; ***$p < 0.001$; ****$p < 0.0001$ (one-way and two-way ANOVA, Tukey's multiple comparison test).

or *in vivo* purposes, respectively. Samples were concentrated in an Amicon centrifugal filters of 50,000 MWCO (Merck KGaA, Darmstadt, Germany) at 2,000×*g* for 5 min per fraction. The final solution was filter sterilized, stored at 4°C and used within 3 days. Encapsulation efficiency and encapsulated mRNA concentration were determined using the RiboGreen RNA Assay Kit (Thermo Fisher Scientific, Waltham, MA, USA).[53]

The mean hydrodynamic size and distribution (PDI) of the LNP formulations were measured by dynamic light scattering in a Nano ZS Zetasizer (Malvern Instruments Corp., Malvern, UK) LNPs loaded with OVA or Luc-mRNA (EE >90%) and mean diameter in the range of 90–100 nm with low PDI (<0.05) were successfully obtained (Table S1).

Mouse NPCs

For the adjuvant testing and co-culture studies liver perfusion was used as method for NPCs isolation. Ten-week-old mice were anesthetized (i.p., 100 mg/kg ketamine and 5 mg/kg xylazine) and the livers were perfused via the portal vein with 20 mL perfusion medium (Ca^{2+}- and Mg^{2+}-free HBSS, Thermo Fisher Scientific) containing 100 U/L collagenase (Sigma-Aldrich, Burlington, MA, USA), 5% heat-inactivated fetal bovine serum (FBS; Gibco, Grand Island, NY, USA) and 10 μg/mL DNase I (Sigma-Aldrich). The livers were dissected and incubated in 5 mL perfusion medium at 37°C for 15 min. Afterward, the livers were teased through 70 μm nylon cell strainers and washed with 50 mL wash medium (RPMI 1640 medium supplemented with 5% FBS, and 100 U/mL penicillin and 100 μg/mL streptomycin purchased from Sigma-Aldrich), and centrifuged at 30×*g*, 4°C for 15 min to pellet the parenchymal cells. The supernatants were collected and centrifuged at 300×*g*, 4°C for 10 min to pellet the NPCs. Cells in the pellet were re-suspended in ice-cold 30% Histodenz (Sigma-Aldrich) in Ca^{2+}- and Mg^{2+}-free HBSS, overlaid with cold HBSS, and centrifuged at 1,500×*g* at 4°C for 20 min without braking during deceleration. The NPCs were collected at the HBSS/Histodenz interface, washed with 12 mL wash medium and centrifuged at 300×*g* at 4°C for 10 min. The cells in the pellet were resuspended in 1 mL RPMI 1640 culture medium (Life Technologies Ltd.; Scotland, UK) containing 10% FBS, 1% L-glutamine, 1% HEPES, and 1% essential and nonessential amino acids, 1% Na-pyruvate, 100 U/mL penicillin, 100 μg/mL streptomycin and 50 μM 2-mercaptoethanol (Gibco), counted, and cultured as described below.

The liver dissociation kit (Miltenyi Biotec, Bergisch Gladbach, Germany) was used to isolate NPCs for *ex vivo* studies. Dissociation mix was prepared in accordance with the supplier's protocol. The livers were dissected from euthanized mice, rinsed with DMEM (Thermo Fisher Scientific), cut into small pieces and transferred into gentleMACS C tubes containing dissociation mix. The tubes were then attached to the gentleMACS dissociator and the liver dissociation program was run. After the program terminated, the samples were re-suspended and transferred to 50-mL Falcon tubes by passing through a 100-μm nylon cell strainer. The samples were centrifuged at 300×*g* at 4°C for 10 min. Supernatants were aspirated, and the red blood cells (RBCs) were lysed by treating with 2 mL RBC lysis buffer (Thermo Fisher Scientific) at room temperature (RT) for 3 min. The RBC-free cells were centrifuged at 400×*g* at 4°C for 10 min. The supernatants were aspirated, and the cell pellets were re-suspended in 1 mL HBSS. The cells were then mixed with 2 mL freshly prepared 30% Histodenz, 1 mL cold HBSS was layered on top, and the samples were centrifuged at 1,500×*g* at 4°C for 20 min without braking during deceleration. Cells at the Histodenz interface were collected, washed, and centrifuged at 300×*g* at 4°C for 10 min. Cells in the pellet were resuspended in culture medium and counted.

NPC stimulation *in vitro*

The NPCs were transferred to 96-well flat-bottom plate containing RPMI 1640 culture medium supplemented with 10% FBS, 1% L-glutamine, 1% HEPES, 1% essential and nonessential amino acids, 1% Na-pyruvate, 100 U/mL penicillin, 100 μg/mL streptomycin, and 50 μM 2-mercaptoethanol (400,000 cells/200 μL) and incubated overnight with 1 μg/mL bacterial endotoxin (LPS) or 5 μg/mL R848, Poly I:C or cGAMP, when combined 10 μg/mL final adjuvant concentration (InvivoGen, San Diego, CA, USA) at 37°C and 5% CO_2.

T cell proliferation

NPCs were transferred to 96-well U-bottom plates (400,000 cells/100 μL) and incubated with 5 μg/mL OVA; after 3 h, 5 μg/mL R848, Poly I:C or cGAMP (InvivoGen) was added and NPCs were incubated at 37°C in 5% CO_2. On the following day, cell samples were collected, washed, resuspended in culture medium, and transferred to triplicate wells in 96-well plates (Greiner Bio-One). Splenic $CD8^+$ T cells specific for $OVA_{257–264}$ peptide in the context of H-$2K^b$, and $CD4^+$ T cells specific for $OVA_{323-339}$ peptide in the context of H-2 I-A^b and I-A^d were isolated by immunomagnetic sorting (Miltenyi Biotec) from OT-I and OT-II mice, respectively, and cultured at 5×10^4 cells/100 μL/well with the NPCs. After 3 days, 10 μL culture supernatant was collected from each well and stored at −20°C for subsequent CBA analysis. ^3H-thymidine (0.5 μCi/well) was added during the last 18 h incubation. Subsequently, cell lysates were transferred onto glass fiber filter mats (Harvester 96; TomTec, Hamden, CT, USA), and the incorporated radioactivity was quantified using a microplate β-counter (1450 MicroBeta Trilux; PerkinElmer, Waltham, MA, USA).

NPC activation *in vivo*

Nine groups of wildtype C57BL/6JRj mice (n = 5) were injected i.v. with 100 μL of one of the following: (i) 7 μg LNPs-OVA; (ii) 25 μg R848; (iii) 5 μg cGAMP; (iv) 7 μg LNPs-OVA and 25 μg R848; (v) 7 μg LNPs-OVA and 5 μg cGAMP; (vi) 7 μg LNPs-OVA, 25 μg R848 and 5 μg cGAMP; (vii) 25 μg R848 and 5 μg cGAMP; or (viii) PBS control. Whole blood was collected on the day following injection by intracardiac puncture under anesthesia (i.p., 100 mg/kg ketamine and 5 mg/kg xylazine) and the mice were euthanized by cervical dislocation. The livers and spleens were dissected. Single cell suspensions were prepared and cultured as described above. A 150-μL aliquot of the supernatant was collected from each well and

stored at −20°C for CBA analysis; and the cells were stained and analyzed by flow cytometry.

Biodistribution and protein expression

Four groups of C57BL/6 mice ($n = 5$) were injected *i.v.* with 100 μL of (i) 7 μg LNPs-Luc; (ii) 7 μg LNPs-Luc and 25 μg R848; (iii) 7 μg LNPs-Luc and 5 μg cGAMP; or (iv) 7 μg LNPs-Luc, 25 μg R848 and 5 μg cGAMP. The substrate, luciferin, was injected i.p. after 6 h and the mice were anesthetized with isoflurane mixed with oxygen 5 min later. After an additional 5 min, the anesthetized mice (3 vol. % isoflurane) were monitored by bioluminescence imaging with an IVIS Spectrum CT (PerkinElmer) using a 3- or 5-s exposure time. The mice were euthanized after imaging by cervical dislocation and the organs (heart, lungs, liver, kidneys, spleen, and auxiliary lymph nodes) were dissected, imaged, and weighed. Images were analyzed with Living Image Software (PerkinElmer).

Mouse immunization and single-cell isolation

The immune response generated after repetitive injections with the LNPs-OVA with and without adjuvants was evaluated. Five groups of C57BL/6JRj mice ($n = 4$) were injected i.v., three times, 5 days apart with a final volume of 100 μL. The cohorts were injected as follows: (i) 7 μg LNPs-OVA; (ii) 7 μg LNPs-OVA and 25 μg R848; (iii) 7 μg LNPs-OVA and 5 μg cGAMP; (iv) 7 μg LNPs-OVA, 25 μg R848 and 5 μg cGAMP; and (v) PBS control. One day after the last boost, the animals were anesthetized (i.p., 100 mg/kg ketamine and 5 mg/kg xylazine), weighted and blood was collected by intracardiac puncture and sera was isolated and stored for further serological analysis and liver transaminases quantitation. Mice were then euthanized by cervical dislocation and livers and spleen were dissected for single-cell isolation. Mouse NPCs were isolated as explained before and spleen single-cell suspension was obtained by spleen disaggregation through a 70-μm strainer. After a washing step, splenocyte suspensions were treated with RBC lysis buffer (Thermo Fisher Scientific) for 5 min at RT and counted for seeding and *ex vivo* stimulation. In contrast, NPCs and splenocytes were stained and analyzed by flow cytometry to evaluate the innate immune response by the activation state of the different cell subpopulations after repetitive injections (Figures S6–S8).

Splenocyte specific stimulation

To evaluate the specific-adaptive immune response against OVA after the immunization scheme, splenocytes were seeded into 96-well U-bottom plates (2×10^6 cells/200 μL/well) in RPMI-1640 medium supplemented with 2% L-glutamine, 10% iFBS and 1% penicillin-streptomycin. Cells were then either unstimulated (only culture medium) or stimulated with 2 μg/mL of the MHC I-restricted SIINFEKL or the MHC II-restricted GRAY peptides (JPT Peptide Technologies GmbH, Berlin, Germany) and incubated at 37°C overnight for intracellular cytokine staining (IFN-γ, IL-2, and TNF-α) and 24 h for quantification of the cytokines in supernatants (Figure S9). Splenocytes were also stimulated with 10 μg/mL of OVA protein (InvivoGen) for 48 h to evaluate the $CD4^+$ as well as the $CD8^+$ T cell-specific proliferation by intranuclear staining of Ki-67 and to estimate the specific response of T_{reg} cells upon MHC II-restricted GRAY peptide stimulation (Figure S10).

Flow cytometry

To assess the activation of the NPC subpopulations after the *in vitro* incubation with adjuvants and upon a single i.v. LNPs-OVA injection with and without adjuvants, cells were washed with 150 μL DPBS, 100 μL 0.05 mM EDTA was added, and the cells were detached by incubation on ice for 30 min. The detached cells were collected; single NPC suspensions were washed with washing buffer (2% FBS in DPBS) and Fc receptors were blocked by the addition of CD16/CD32-specific antibody (clone 2.4G2, Invitrogen) at 4°C for 15 min. The cells were stained with fluorescent dye-conjugated monoclonal antibodies (Table S2) specific for: mouse CD45 (eFlour 506) purchased from InvitroGen; F4/80 (AF 488), CD86 (APC) purchased from BioLegend; CD32b (PE), CD11c (PE-Cy7), CD80 (SuperBright 436), and CD86 (APC) purchased from eBioscience (San Diego, CA, USA) for 30 min at 4°C in the dark. The stained cells were washed, and viability was assessed by incubation with 7-AAD (BD Biosciences, Franklin Lakes, NJ, USA) at RT for 5 min in the dark. The cells were analyzed by flow cytometry (Figures S6 and S7) using an LSRII flow cytometer (BD Biosciences) and FlowJo software v10.8.0 (BD Biosciences).

CBA

Cell culture supernatants were collected and stored at −20°C prior to cytokine/chemokine analysis. The cytokine and chemokine concentrations were quantified using the LEGENDplex mouse anti-virus response panel kit, 13-plex(BioLegend) that includes the following murine cytokines/chemokines: IFN-γ, KC, TNF-α, MCP-1, IL-12p70, RANTES, IL-1β, IP-10, GM-CSF, IL-10, IFN-β, IFN-α, and IL-6. The assay was performed as recommended by the manufacturer (BioLegend). Samples were acquired with an LSRII flow cytometer (BD Biosciences). Results were analyzed using Qognit Legendplex Analysis Software (Biolegend). The cytokines released from the 24 h peptide-stimulated splenocytes isolated in the immunization study were quantified using the LEGENDplex mouse Th Cytokine Panel kit (BioLegend) including IFN-γ, TNF-α, IL-2, IL-6, IL-10, IL-4, and IL-22. The samples were acquired with a Symphony A3 flow cytometer (BD Biosciences) and the results were analyzed using the Qognit Legendplex Analysis Software (BioLegend).

Serological analysis

The sera were isolated from whole blood in the immunization study and anti-OVA IgG titers were determined by ELISA. Briefly, 96-half area well plates were coated with 10 μg/mL OVA protein at 4°C in humid chamber overnight. Plates were washed, blocked with 3% skim milk-PBS and the sera were incubated in serial dilutions at RT for 1 h. Anti-mouse IgG H&L (HRP) (ab6789; Abcam) was applied as secondary antibody and plates were developed with TMB ELISA substrate and read at 450 nm in an ELISA plate reader after adding Stop solution. Titers were calculated using regression analysis.

Statistical analysis

All graphs and statistical analyses were performed with GraphPad Prism software v10.1.2 (GraphPad Software, San Diego, CA, USA). One-way ANOVA followed by Dunnett's or Tukey's multiple comparison test and two-way ANOVA followed by Tukey's multiple comparison test was used to compare three or more groups. Data are the means ± SEM. Significantly different between groups: ns, not significant; $^{*}p < 0.05$; $^{**}p < 0.01$; $^{***}p < 0.001$; $^{****}p < 0.0001$.

DATA AVAILABILITY

Data supporting the findings of this study are available from the corresponding author, M.L.C., upon request. The data are not publicly available due to restrictions that could compromise proprietary information.

ACKNOWLEDGMENTS

This research was funded by Deutsche Forschungsgemeinschaft (DFG), grant number SFB1066-3 and the subprojects B15 (M.S. and S.G.) and B17 (P.S. and L.K.). The animal study protocols were approved by the local authorities (Landesuntersuchungsamt Rhineland-Palatinate). Ethical approval was granted by the Landesuntersuchungsamt LUA, Koblenz, AK G20-1-123 and G20-1-130. All graphical abstract and figure schemes were created with Biorender software (https://BioRender.com/d35a436). The authors are grateful to Stephen H. Gregory (Providence, RI, USA) for editing this manuscript.

AUTHOR CONTRIBUTIONS

M.L.C., S.G., M.B., and M.J.L. designed and planned experiments. L.K. provided oversight on the study design. M.S. and M.J.L. performed *in vitro* and *in vivo* experiments. K.H., S.F.E., and R.G. assisted with *in vitro* experiments. R.G., L.P., Y.Z., and P.S. assisted with *in vivo* experiments. G.A.I. and I.R.B. prepared LNP formulations. M.S. and M.J.L. processed animal samples and tissues. M.S. and M.J.L. analyzed data and performed statistical analyses. M.S. and M.J.L. drafted figures. M.L.C. wrote and edited the manuscript. S.G. and M.B. provided financial support for the study. All authors performed critical revision and approved final manuscript text and figures.

DECLARATION OF INTERESTS

M.L.C. is currently an employee at BioNTech SE (Mainz, Germany); however, the contributions from M.L.C. were made prior to his employment at BioNTech SE. The remaining authors declare no conflict of interest. L.K. declares receiving travel expenses and speaker honoraria from Gilead science (Foster City, CA, USA) and Takeda (Doshomachi, Japan).

SUPPLEMENTAL INFORMATION

Supplemental information can be found online at https://doi.org/10.1016/j.omtm.2025.101427.

REFERENCES

1. Thomson, A.W., and Knolle, P.A. (2010). Antigen-presenting cell function in the tolerogenic liver environment. Nat. Rev. Immunol. *10*, 753–766. https://doi.org/10.1038/nri2858.
2. Cacicedo, M.L., Medina-Montano, C., Kaps, L., Kappel, C., Gehring, S., and Bros, M. (2020). Role of Liver-Mediated Tolerance in Nanoparticle-Based Tumor Therapy. Cells *9*, 1985. https://doi.org/10.3390/cells9091985.
3. Wilkinson, A.L., Qurashi, M., and Shetty, S. (2020). The Role of Sinusoidal Endothelial Cells in the Axis of Inflammation and Cancer Within the Liver. Front. Physiol. *11*, 990. https://doi.org/10.3389/fphys.2020.00990.
4. Losikoff, P.T., Self, A.A., and Gregory, S.H. (2012). Dendritic cells, regulatory T cells and the pathogenesis of chronic hepatitis C. Virulence *3*, 610–620. https://doi.org/10.4161/viru.21823.
5. Heymann, F., Peusquens, J., Ludwig-Portugall, I., Kohlhepp, M., Ergen, C., Niemietz, P., Martin, C., van Rooijen, N., Ochando, J.C., Randolph, G.J., et al. (2015). Liver Inflammation Abrogates Immunological Tolerance Induced by Kupffer Cells. Hepatology *62*, 279–291. https://doi.org/10.1002/hep.27793.
6. Horst, A.K., Neumann, K., Diehl, L., and Tiegs, G. (2016). Modulation of liver tolerance by conventional and nonconventional antigen-presenting cells and regulatory immune cells. Cell. Mol. Immunol. *13*, 277–292. https://doi.org/10.1038/cmi.2015.112.
7. Carambia, A., Freund, B., Schwinge, D., Heine, M., Laschtowitz, A., Huber, S., Wraith, D.C., Korn, T., Schramm, C., Lohse, A.W., et al. (2014). TGF-β-dependent induction of CD4+CD25+Foxp3 + Tregs by liver sinusoidal endothelial cells. J. Hepatol. *61*, 594–599. https://doi.org/10.1016/j.jhep.2014.04.027.
8. Grover, P., Goel, P.N., and Greene, M.I. (2021). Regulatory T Cells: Regulation of Identity and Function. Front. Immunol. *12*, 750542. https://doi.org/10.3389/fimmu.2021.750542.
9. Protzer, U., Maini, M.K., and Knolle, P.A. (2012). Living in the liver: Hepatic infections. Nat. Rev. Immunol. *12*, 201–213. https://doi.org/10.1038/nri3169.
10. Lugade, A.A., Kalathil, S., Miller, A., Iyer, R., and Thanavala, Y. (2013). High immunosuppressive burden in advanced hepatocellular carcinoma patients: Can effector functions be restored? OncoImmunology *2*, e24679. https://doi.org/10.4161/onci.24679.
11. Wang, X., Dong, A., Xiao, J., Zhou, X., Mi, H., Xu, H., Zhang, J., and Wang, B. (2016). Overcoming HBV immune tolerance to eliminate HBsAg-positive hepatocytes via pre-administration of GM-CSF as a novel adjuvant for a hepatitis B vaccine in HBV transgenic mice. Cell. Mol. Immunol. *13*, 850–861. https://doi.org/10.1038/cmi.2015.64.
12. Zheng, M., and Tian, Z. (2019). Liver-Mediated Adaptive Immune Tolerance. Front. Immunol. *10*, 2525. https://doi.org/10.3389/fimmu.2019.02525.
13. Sankar, K., Pearson, A.N., Worlikar, T., Perricone, M.D., Holcomb, E.A., Mendiratta-Lala, M., Xu, Z., Bhowmick, N., and Green, M.D. (2023). Impact of immune tolerance mechanisms on the efficacy of immunotherapy in primary and secondary liver cancers. Transl. Gastroenterol. Hepatol. *8*, 29. https://doi.org/10.21037/tgh-23-11.
14. Schramm, C., Oo, Y.H., and Lohse, A.W. (2022). Tolerance and autoimmunity in the liver. Semin. Immunopathol. *44*, 393–395. https://doi.org/10.1007/s00281-022-00952-6.
15. Su, J., Brunner, L., Ates Oz, E., Sacherl, J., Frank, G., Kerth, H.A., Thiele, F., Wiegand, M., Mogler, C., Aguilar, J.C., et al. (2023). Activation of CD4 T cells during prime immunization determines the success of a therapeutic hepatitis B vaccine in HBV-carrier mouse models. J. Hepatol. *78*, 717–730. https://doi.org/10.1016/j.jhep.2022.12.013.
16. Kaps, L., Limeres, M.J., Schneider, P., Svensson, M., Zeyn, Y., Fraude, S., Cacicedo, M.L., Galle, P.R., Gehring, S., and Bros, M. (2023). Liver Cell Type-Specific Targeting by Nanoformulations for Therapeutic Applications. Int. J. Mol. Sci. *24*, 11869. https://doi.org/10.3390/ijms241411869.
17. Zhao, T., Cai, Y., Jiang, Y., He, X., Wei, Y., Yu, Y., and Tian, X. (2023). Vaccine adjuvants: mechanisms and platforms. Signal Transduct. Targeted Ther. *8*, 283. https://doi.org/10.1038/s41392-023-01557-7.
18. Ong, G.H., Lian, B.S.X., Kawasaki, T., and Kawai, T. (2021). Exploration of Pattern Recognition Receptor Agonists as Candidate Adjuvants. Front. Cell. Infect. Microbiol. *11*, 745016. https://doi.org/10.3389/fcimb.2021.745016.
19. Kutscher, S., Bauer, T., Dembek, C., Sprinzl, M., and Protzer, U. (2012). Design of therapeutic vaccines: Hepatitis B as an example. Microb. Biotechnol. *5*, 270–282. https://doi.org/10.1111/j.1751-7915.2011.00303.x.
20. Kowalzik, F., Schreiner, D., Jensen, C., Teschner, D., Gehring, S., and Zepp, F. (2021). Mrna-based vaccines. Vaccines *9*, 390. https://doi.org/10.3390/vaccines9040390.
21. Cacicedo, M.L., Limeres, M.J., and Gehring, S. (2022). mRNA-Based Approaches to Treating Liver Diseases. Cells *11*, 3328. https://doi.org/10.3390/cells11203328.
22. Beck, J.D., Reidenbach, D., Salomon, N., Sahin, U., Türeci, Ö., Vormehr, M., and Kranz, L.M. (2021). mRNA therapeutics in cancer immunotherapy. Mol. Cancer *20*, 69. https://doi.org/10.1186/s12943-021-01348-0.
23. Lee, Y., Jeong, M., Park, J., Jung, H., and Lee, H. (2023). Immunogenicity of lipid nanoparticles and its impact on the efficacy of mRNA vaccines and therapeutics. Exp. Mol. Med. *55*, 2085–2096. https://doi.org/10.1038/s12276-023-01086-x.
24. Verbeke, R., Hogan, M.J., Loré, K., and Pardi, N. (2022). Innate immune mechanisms of mRNA vaccines. Immunity *55*, 1993–2005. https://doi.org/10.1016/j.immuni.2022.10.014.

25. Xie, C., Yao, R., and Xia, X. (2023). The advances of adjuvants in mRNA vaccines. NPJ Vaccines *8*, 162. https://doi.org/10.1038/s41541-023-00760-5.

26. Shan, F., Somasundaram, A., Bruno, T.C., Workman, C.J., and Vignali, D.A.A. (2022). Therapeutic targeting of regulatory T cells in cancer. Trends Cancer *8*, 944–961. https://doi.org/10.1016/j.trecan.2022.06.008.

27. Gutjahr, A., Papagno, L., Nicoli, F., Kanuma, T., Kuse, N., Cabral-Piccin, M.P., Rochereau, N., Gostick, E., Lioux, T., Perouzel, E., et al. (2019). The STING ligand cGAMP potentiates the efficacy of vaccine-induced CD8+ T cells. JCI Insight *4*, e125107. https://doi.org/10.1172/jci.insight.125107.

28. Ren, H., Jia, W., Xie, Y., Yu, M., and Chen, Y. (2023). Adjuvant physiochemistry and advanced nanotechnology for vaccine development. Chem. Soc. Rev. *52*, 5172–5254. https://doi.org/10.1039/d2cs00848c.

29. Frega, G., Wu, Q., Le Naour, J., Vacchelli, E., Galluzzi, L., Kroemer, G., and Kepp, O. (2020). Trial Watch: experimental TLR7/TLR8 agonists for oncological indications. OncoImmunology *9*, 1796002. https://doi.org/10.1080/2162402X.2020.1796002.

30. Lu, R., Groer, C., Kleindl, P.A., Moulder, K.R., Huang, A., Hunt, J.R., Cai, S., Aires, D.J., Berkland, C., and Forrest, M.L. (2019). Formulation and preclinical evaluation of a toll-like receptor 7/8 agonist as an anti-tumoral immunomodulator. J. Contr. Release *306*, 165–176. https://doi.org/10.1016/j.jconrel.2019.06.003.

31. Liu, J., Jiang, M., Ma, Z., Dietze, K.K., Zelinskyy, G., Yang, D., Dittmer, U., Schlaak, J.F., Roggendorf, M., and Lu, M. (2013). TLR1/2 Ligand–Stimulated Mouse Liver Endothelial Cells Secrete IL-12 and Trigger CD8+ T Cell Immunity In Vitro. J. Immunol. *191*, 6178–6190. https://doi.org/10.4049/jimmunol.1301262.

32. Pulendran, B., S Arunachalam, P., and O'Hagan, D.T. (2021). Emerging concepts in the science of vaccine adjuvants. Nat. Rev. Drug Discov. *20*, 454–475. https://doi.org/10.1038/s41573-021-00163-y.

33. Cheng, F., Su, T., Zhou, S., Liu, X., Yang, S., Lin, S., Guo, W., and Zhu, G. (2023). Single-dose injectable nanovaccine-in-hydrogel for robust immunotherapy of large tumors with abscopal effect. Sci. Adv. *9*, eade6257. https://doi.org/10.1126/sciadv.ade6257.

34. Hänel, G., Angerer, C., Petry, K., Lichtenegger, F.S., and Subklewe, M. (2022). Blood DCs activated with R848 and poly(I:C) induce antigen-specific immune responses against viral and tumor-associated antigens. Cancer Immunol. Immunother. *71*, 1705–1718. https://doi.org/10.1007/s00262-021-03109-w.

35. Anfray, C., Varela, C.F., Ummarino, A., Maeda, A., Sironi, M., Gandoy, S., Brea, J., Loza, M.I., León, S., Calvo, A., et al. (2023). Polymeric nanocapsules loaded with poly(I:C) and resiquimod to reprogram tumor-associated macrophages for the treatment of solid tumors. Front. Immunol. *14*, 1334800. https://doi.org/10.3389/fimmu.2023.1334800.

36. Chung, S., Lee, C.M., and Zhang, M. (2022). Advances in nanoparticle-based mRNA delivery for liver cancer and liver-associated infectious diseases. Nanoscale Horiz. *8*, 10–28. https://doi.org/10.1039/d2nh00289b.

37. González-García, L.E., Macgregor, M.N., Visalakshan, R.M., Lazarian, A., Cavallaro, A.A., Morsbach, S., Mierczynska-Vasilev, A., Mailänder, V., Landfester, K., and Vasilev, K. (2022). Nanoparticles Surface Chemistry Influence on Protein Corona Composition and Inflammatory Responses. Nanomaterials *12*, 682. https://doi.org/10.3390/nano12040682.

38. Zhu, W., Wei, L., Dong, C., Wang, Y., Kim, J., Ma, Y., Gonzalez, G.X., and Wang, B.Z. (2022). cGAMP-adjuvanted multivalent influenza mRNA vaccines induce broadly protective immunity through cutaneous vaccination in mice. Mol. Ther. Nucleic Acids *30*, 421–437. https://doi.org/10.1016/j.omtn.2022.10.024.

39. Zhou, L., Yi, W., Zhang, Z., Shan, X., Zhao, Z., Sun, X., Wang, J., Wang, H., Jiang, H., Zheng, M., et al. (2023). STING agonist-boosted mRNA immunization via intelligent design of nanovaccines for enhancing cancer immunotherapy. Natl. Sci. Rev. *10*, nwad214. https://doi.org/10.1093/nsr/nwad214.

40. Hejdankova, Z., Vanek, V., Sedlak, F., Prochazka, J., Diederichs, A., Kereïche, S., Novotna, B., Budesinsky, M., Birkus, G., Grantz Saskova, K., and Cigler, P. (2021). Lipid Nanoparticles for Broad-Spectrum Nucleic Acid Delivery. Adv. Funct. Mater. *31*, 2101391. https://doi.org/10.1002/adfm.202101391.

41. Kobiyama, K., and Ishii, K.J. (2022). Making innate sense of mRNA vaccine adjuvanticity. Nat. Immunol. *23*, 474–476. https://doi.org/10.1038/s41590-022-01168-4.

42. Zhuang, X., Chen, L., Yang, S., Xia, S., Xu, Z., Zhang, T., Zeng, B., Yu, T., Yu, N., Wang, W., et al. (2022). R848 Adjuvant Laden With Self-Assembled Nanoparticle-Based mRNA Vaccine Elicits Protective Immunity Against H5N1 in Mice. Front. Immunol. *13*, 836274. https://doi.org/10.3389/fimmu.2022.836274.

43. Islam, M.A., Rice, J., Reesor, E., Zope, H., Tao, W., Lim, M., Ding, J., Chen, Y., Aduluso, D., Zetter, B.R., et al. (2021). Adjuvant-pulsed mRNA vaccine nanoparticle for immunoprophylactic and therapeutic tumor suppression in mice. Biomaterials *266*, 120431. https://doi.org/10.1016/j.biomaterials.2020.120431.

44. Rojas, L.A., Sethna, Z., Soares, K.C., Olcese, C., Pang, N., Patterson, E., Lihm, J., Ceglia, N., Guasp, P., Chu, A., et al. (2023). Personalized RNA neoantigen vaccines stimulate T cells in pancreatic cancer. Nature *618*, 144–150. https://doi.org/10.1038/s41586-023-06063-y.

45. Sahin, U., Derhovanessian, E., Miller, M., Kloke, B.P., Simon, P., Löwer, M., Bukur, V., Tadmor, A.D., Luxemburger, U., Schrörs, B., et al. (2017). Personalized RNA mutanome vaccines mobilize poly-specific therapeutic immunity against cancer. Nature *547*, 222–226. https://doi.org/10.1038/nature23003.

46. Zinkernagel, R.M., Lamarre, A., Ciurea, A., Hunziker, L., Ochsenbein, A.F., Mccoy, K.D., Fehr, T., Bachmann, M.F., Kalinke, U., and Hengartner, H. (2001). Neutralizing Antiviral Antibody Responses. Adv. Immunol. *79*, 1–53. https://doi.org/10.1016/S0065-2776(01)79001-3.

47. Billeskov, R., Beikzadeh, B., and Berzofsky, J.A. (2019). The effect of antigen dose on T cell-targeting vaccine outcome. Hum. Vaccines Immunother. *15*, 407–411. https://doi.org/10.1080/21645515.2018.1527496.

48. Dąbrowska, A., Grubba, M., Balihodzic, A., Szot, O., Sobocki, B.K., and Perdyan, A. (2023). The Role of Regulatory T Cells in Cancer Treatment Resistance. Int. J. Mol. Sci. *24*, 14114. https://doi.org/10.3390/ijms241814114.

49. Zhou, J., Xu, Y., Wang, G., Mei, T., Yang, H., and Liu, Y. (2022). The TLR7/8 agonist R848 optimizes host and tumor immunity to improve therapeutic efficacy in murine lung cancer. Int. J. Oncol. *61*, 81. https://doi.org/10.3892/IJO.2022.5371.

50. Chaudhary, B., and Elkord, E. (2016). Regulatory T cells in the tumor microenvironment and cancer progression: Role and therapeutic targeting. Vaccines *4*, 28. https://doi.org/10.3390/vaccines4030028.

51. Wang, Z., Jacobus, E.J., Stirling, D.C., Krumm, S., Flight, K.E., Cunliffe, R.F., Mottl, J., Singh, C., Mosscrop, L.G., Santiago, L.A., et al. (2023). Reducing cell intrinsic immunity to mRNA vaccine alters adaptive immune responses in mice. Mol. Ther. Nucleic Acids *34*, 102045. https://doi.org/10.1016/j.omtn.2023.102045.

52. Verbeke, R., Lentacker, I., Wayteck, L., Breckpot, K., Van Bockstal, M., Descamps, B., Vanhove, C., De Smedt, S.C., and Dewitte, H. (2017). Co-delivery of nucleoside-modified mRNA and TLR agonists for cancer immunotherapy: Restoring the immunogenicity of immunosilent mRNA. J. Contr. Release *266*, 287–300. https://doi.org/10.1016/j.jconrel.2017.09.041.

53. Gambaro, R., Rivero Berti, I., Limeres, M.J., Huck-Iriart, C., Svensson, M., Fraude, S., Pretsch, L., Si, S., Lieberwirth, I., Gehring, S., et al. (2024). Optimizing mRNA-Loaded Lipid Nanoparticles as a Potential Tool for Protein-Replacement Therapy. Pharmaceutics *16*, 771. https://doi.org/10.3390/pharmaceutics.

OMTM, Volume *33*

Supplemental information

mRNA-LNP vaccine strategies: Effects of adjuvants on non-parenchymal liver cells and tolerance

Malin Svensson, María José Limeres, Yanira Zeyn, Rocio C. Gambaro, German A. Islan, Ignacio Rivero Berti, Silvia Fraude-El Ghazi, Leah Pretsch, Katja Hilbert, Paul Schneider, Leonard Kaps, Matthias Bros, Stephan Gehring, and Maximiliano L. Cacicedo

Supplemental Material

Table S1: Characterization of Genvoy-ILM^{TM} LNPs by dynamic light scattering (DLS) and quantification of encapsulation efficiency (EE) by modified Ribogreen® assay.

Formulation	Mean size (nm)	PDI index	Z potential (mV)	EE (%)
OVA mRNA-LNPs	93.4 ± 0.7	0.051 ± 0.009	-2.7 ± 0.5	85.0 ± 2.0
Luc mRNA-LNPs	102.4 ± 2.1	0.063 ± 0.010	-2.0 ± 0.5	93.1 ± 1.5

PDI: polydispersity index

Table S2: Inventory of antibodies applied for flow cytometry analysis.

Marker	Fluorochrome	Clone	Reference	Dilution
Characterization of NPC subpopulations				
F4/80	Alexa Fluor 488	BM8	123120	1/50
CD32b	PE	AT130-2	12-0321-82	1/50
CD11c	PE-Cy7	N418	117318	1/80
CD45	eFluor 506	30-F11	69-0451-82	1/40
CD80	Super Bright 436	16-10A1	62-0801-82	1/333
CD86	APC	GL-1	105012	1/133
Viability dye	7-AAD	-	559925	1/25
Activation state of the NPC subpopulations (immunization study)				
F4/80	Alexa Fluor 488	BM8	123120	1/50
CD32b	PE	AT130-2	12-0321-82	1/50
CD11c	BV650	N418	117339	1/80
CD45	BUV395	30-F11	363-0451-82	1/200
CD80	BUV563	16-10A1	741272	1/40
CD86	BV421	GL-1	105032	1/80
MHC-II (I-A/I-E)	APC-eFluor 780	M5/114.15.2	47-5321-82	1/320

Viability dye	7-AAD	-	559925	1/25
T cell stimulation by intracellular cytokine staining				
CD3	eFluor 506	17A2	69-0032-82	1/66
CD4	FITC	L3T4	553047	1/833
CD8α	Super Bright 436	53-6.7	62-0081-82	1/125
IL-2	PE	JES6-5H4	554428	1/80
IFN-γ	APC	XMG1.2	554413	1/166
TNF-α	PE-Cy7	MP6-XT22	25-7321-82	1/166
Viability dye	L/D Fix Near-IR	-	L34975	0.5:1000
T cell proliferation and Treg response by intranuclear staining				
CD3	eFluor 506	17A2	69-0032-82	1/66
CD4	FITC	L3T4	553047	1/833
CD8α	Super Bright 436	53-6.7	62-0081-82	1/125
CD25	PE-Cy7	PC61.5	25-0251-82	1/200
FoxP3	APC	FJK-16s	17-5773-82	1/20
Ki-67	PE	REA183	130-120-417	1/50
Viability dye	L/D Fix Near-IR	-	L34975	0.5:1000

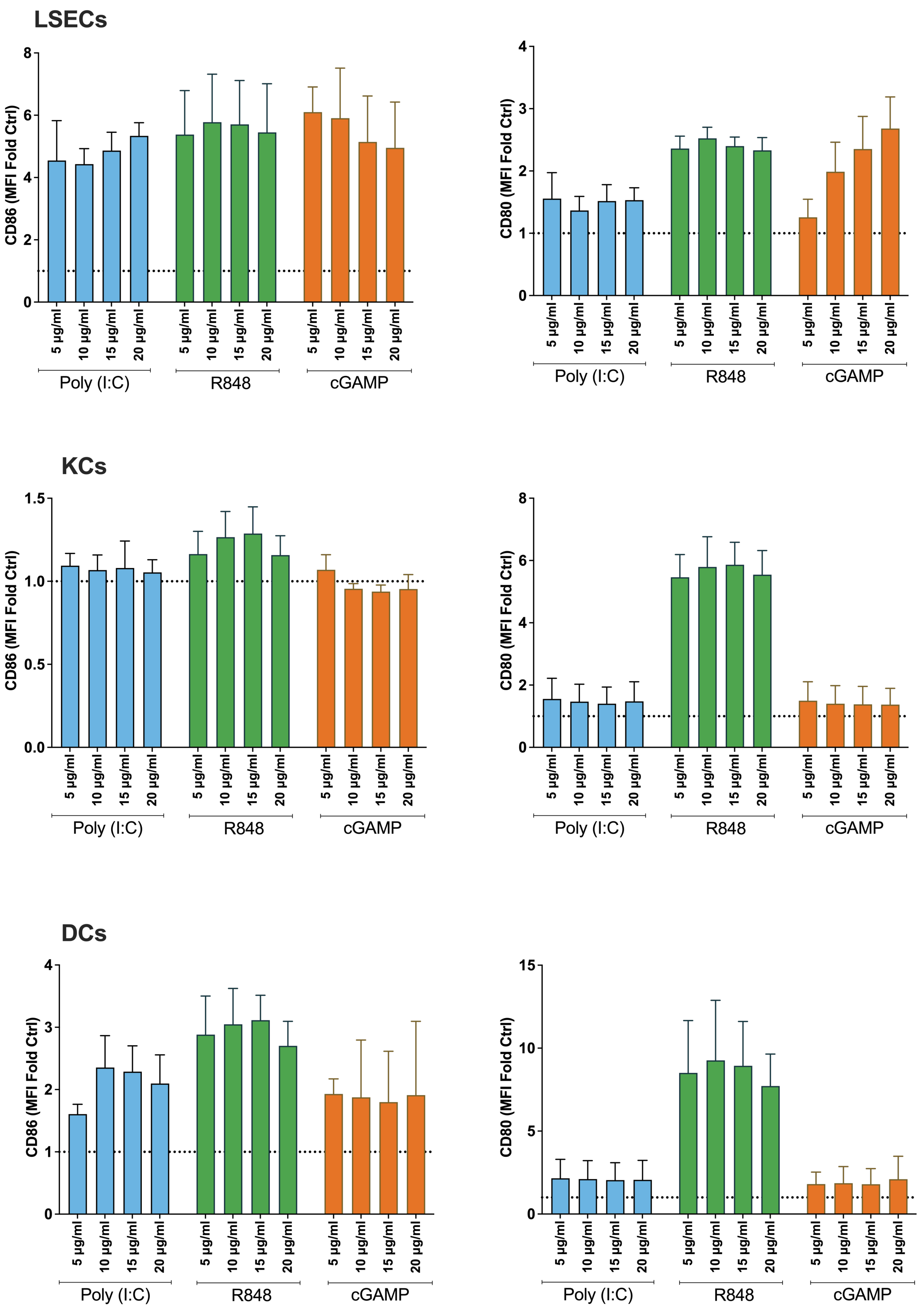

Figure S1. Adjuvants stimulate the expression of CD86 and CD80 by NPC subpopulations. Dose titration was performed in NPC cultures with 5 to 20 µg/mL R848, Poly I:C or cGAMP. CD86 and CD80 expression was evaluated by flow cytometry in Kupffer cells (KCs), dendritic cells (DCs) and Liver sinusoidal endothelial cells (LSECs). Data are the means ± SEM (n = 3).

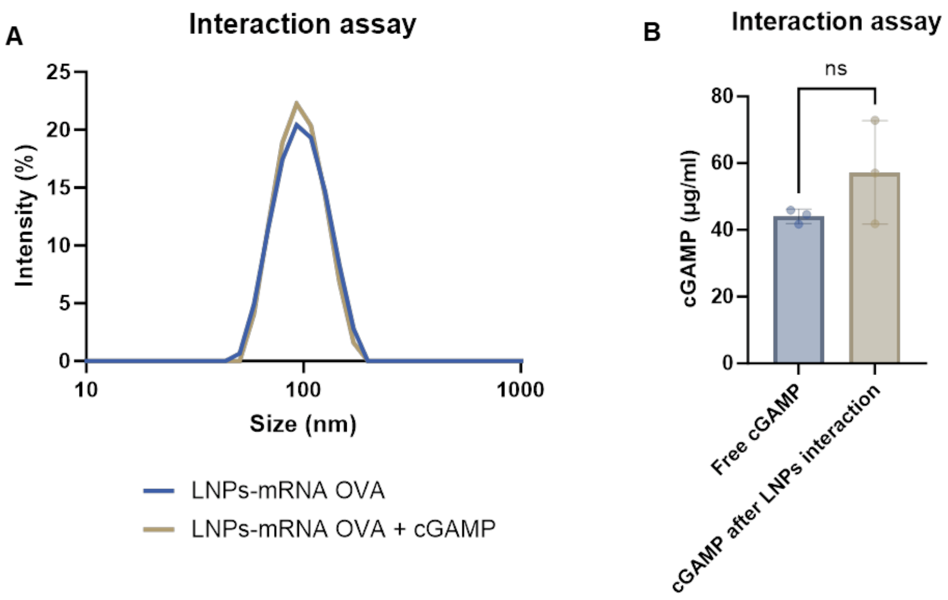

Figure S2. Interaction assay between LNPs-mRNA OVA and cGAMP. 60 µL of LNPs-mRNA OVA containing 7 µg of mRNA were mixed with 10 µL (5 µg) of cGAMP. The volume was adjusted to 100 µL with PBS. Controls of LNPs without cGAMP and free cGAMP were performed. The mixture was left interacting for 10 min at 25°C. After incubation, the mean size of the LNPs was measured by DLS. Then, 500 µL of the mixture were transferred to a centrifugal device (Microcon® centrifugal filters 100 kDa, Merck Millipore, Billerica, MA, USA) and the concentration of cGAMP was determined in the filtrate by ELISA.

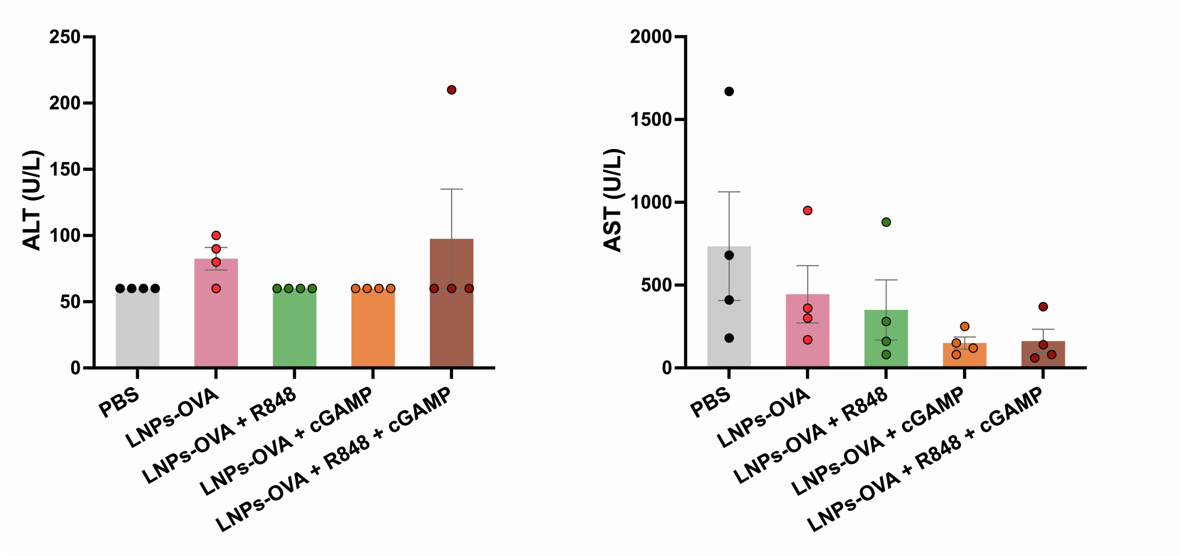

Figure S3. Serum alanine transaminase (ALT) and aspartate transaminase (AST) at termination of the immunization study. Data are mean ± SEM (n = 4). Non-significant differences were found between groups (one-way ANOVA, Tukey´s multiple comparison test).

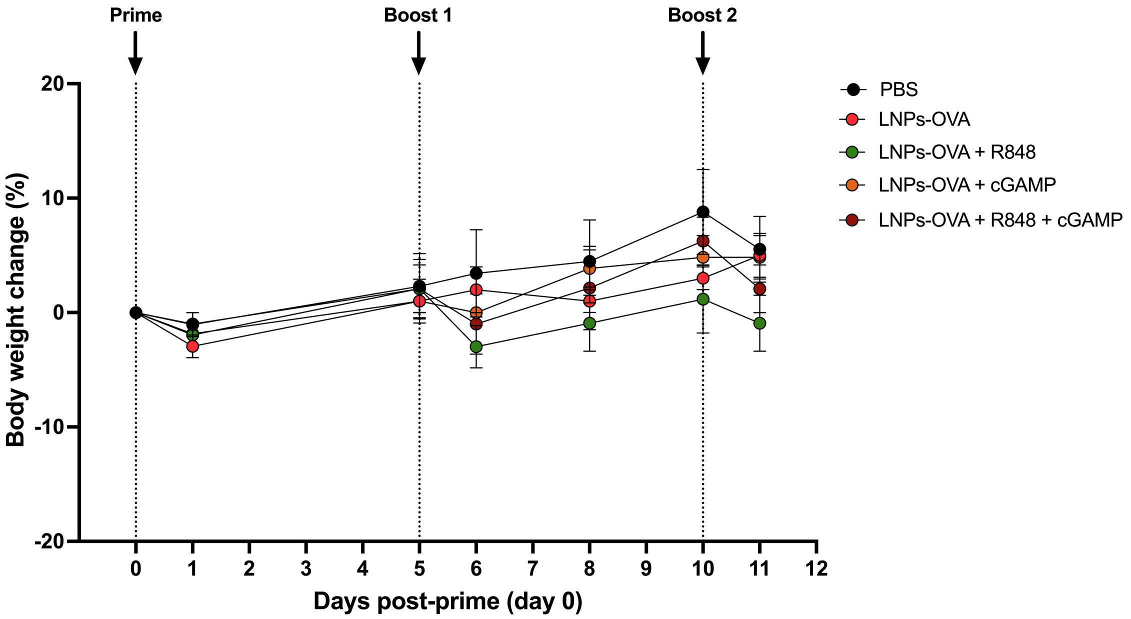

Figure S4. Mice body weight change of the immunization study. The reactogenicity of the LNP formulations plus adjuvants was measured by calculation of the body weight change of the immunized mice.

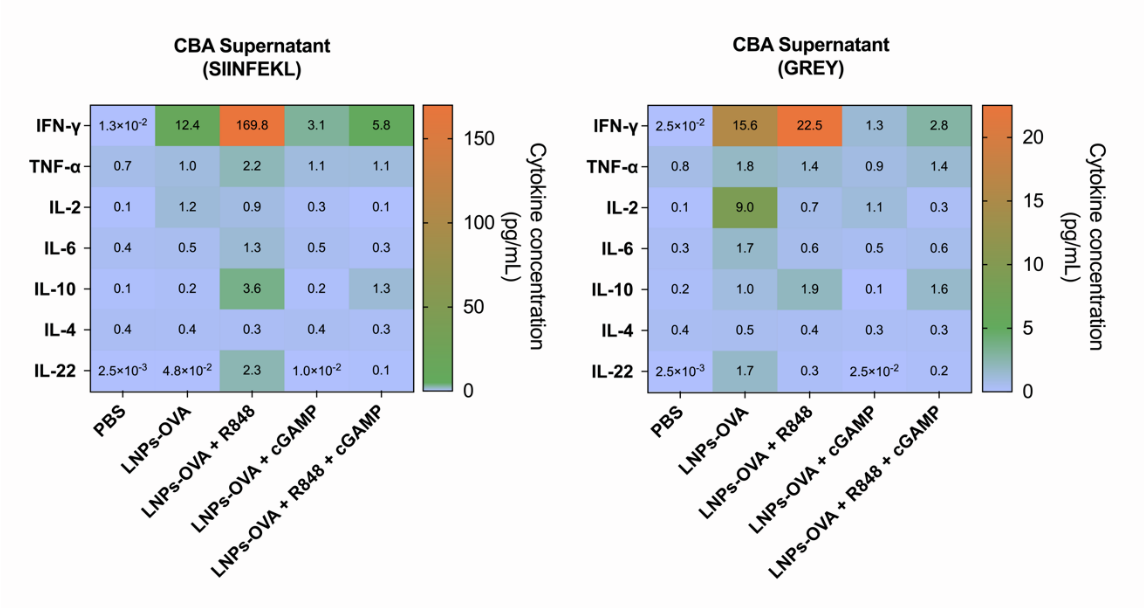

Figure S5. Cytokine quantification in supernatant of 24h-peptide-stimulated splenocytes. Single-cell spleen suspensions from immunized mice were seeded and stimulated with SIINFEKL and GREY peptides for 24h. Supernatants were collected, and the cytokine concentrations were determined using the LEGENDplex mouse Th Cytokine Panel (BioLegend).

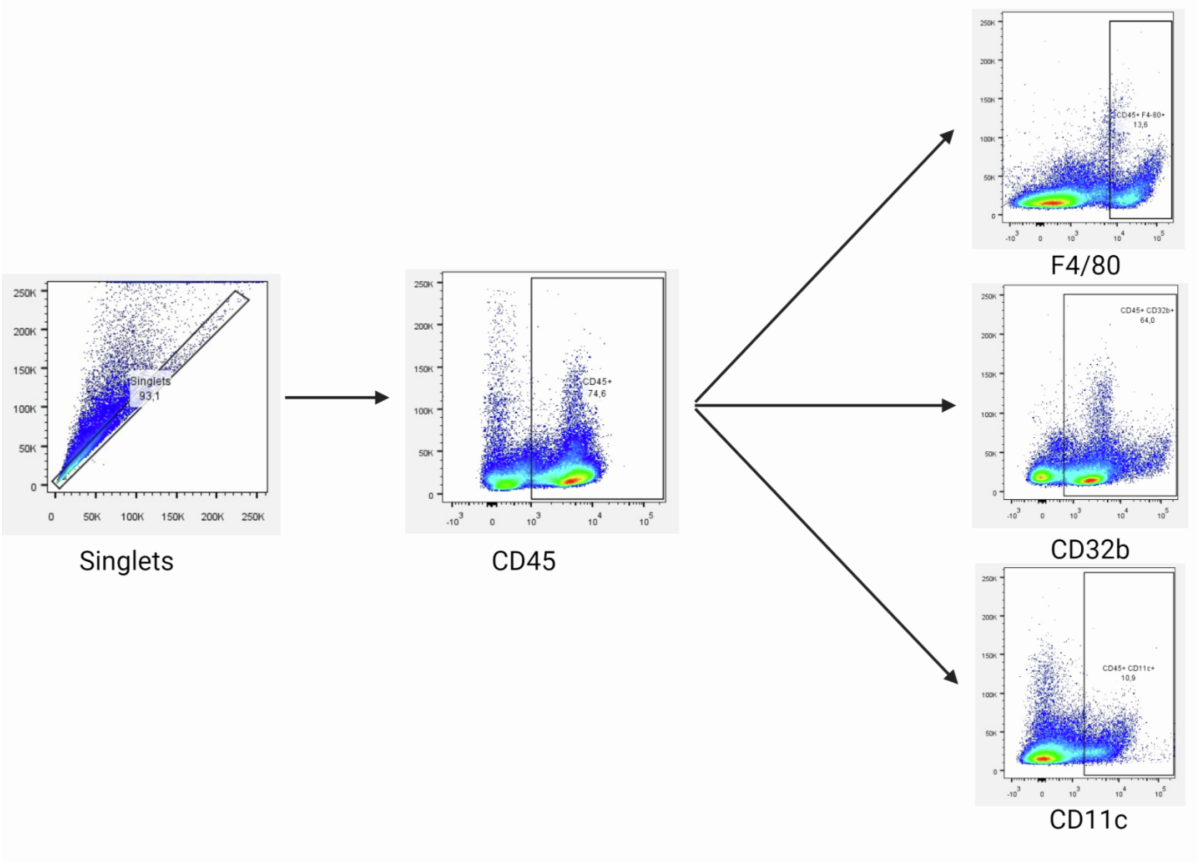

Figure S6. Gating strategy for the different viable NPC subpopulations. Kupffer cells (KCs): CD45$^+$ F4/80$^+$; Liver sinusoidal endothelial cells (LSECs): CD45$^+$ CD32b$^+$; Dendritic cells (DCs): CD45$^+$ CD11c$^+$.

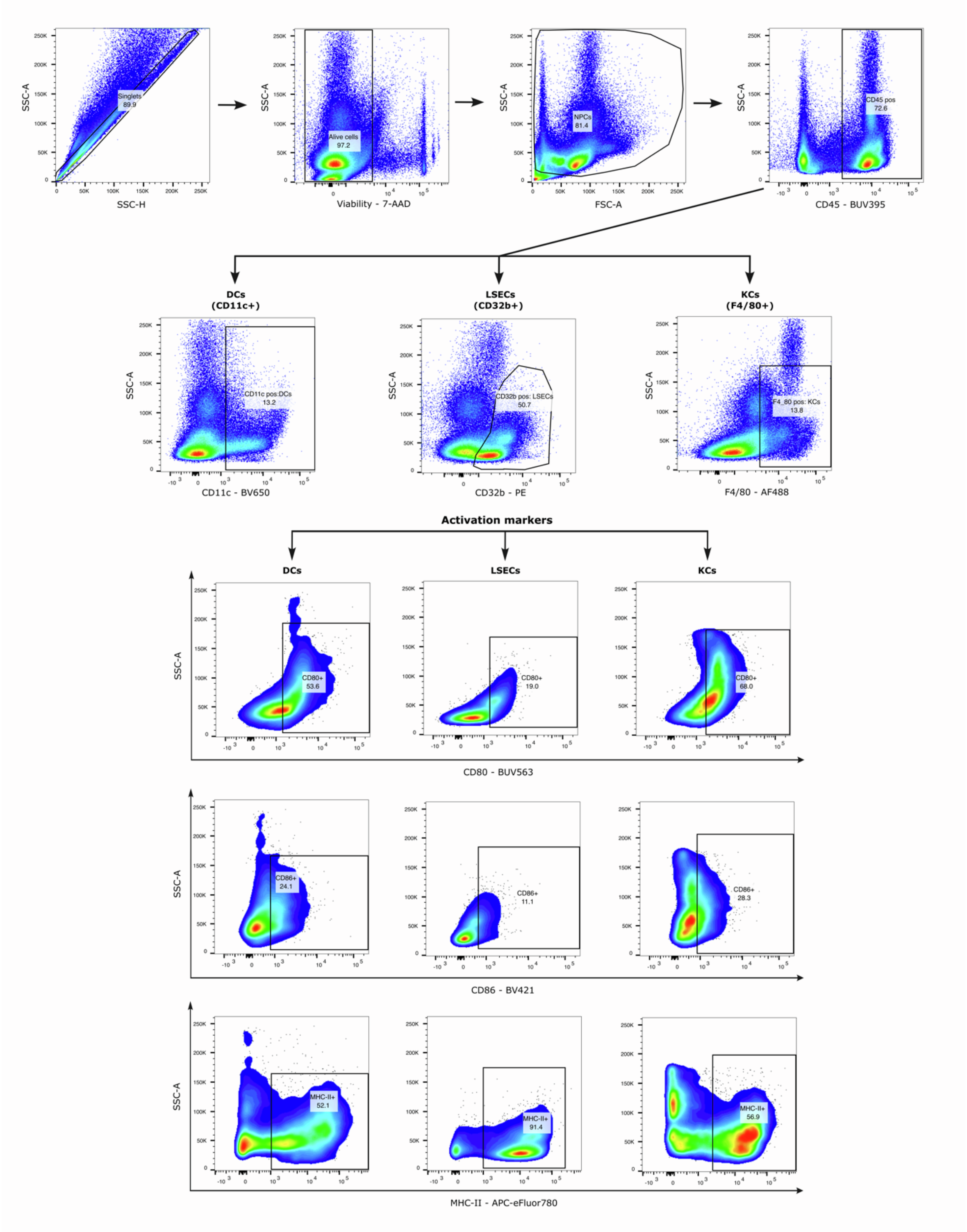

Figure S7. Gating strategy for the activation state (CD80, CD86 and MHC-II) of the different NPC subpopulations of the immunization study. Dendritic cells (DCs): $CD45^+$ $CD11c^+$; Liver sinusoidal endothelial cells (LSECs): $CD45^+$ $CD32b^+$; Kupffer cells (KCs): $CD45^+$ $F4/80^+$.

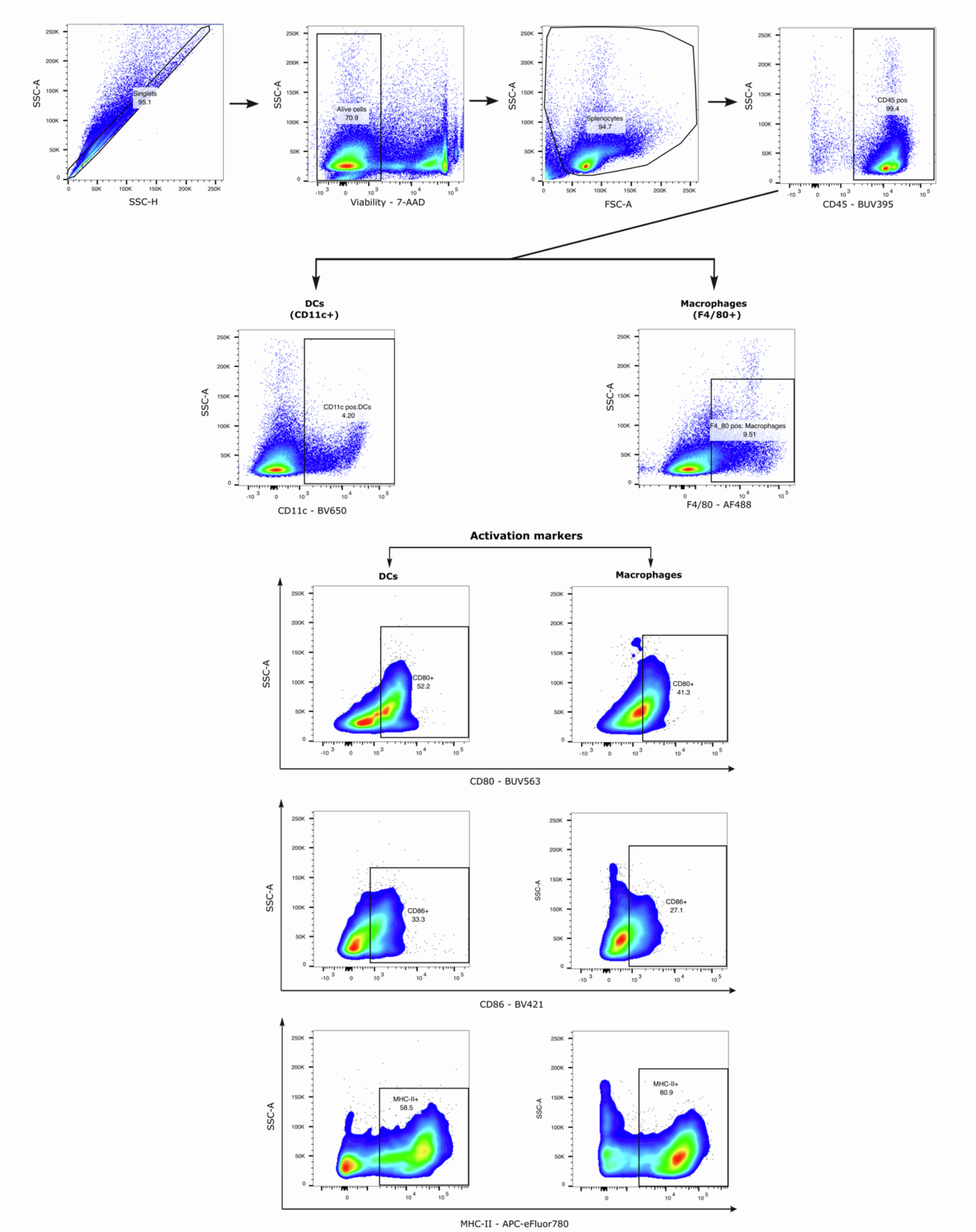

Figure S8. Gating strategy for the activation state (CD80, CD86 and MHC-II) of the different splenocyte subpopulations of the immunization study. Dendritic cells (DCs): CD45^{+} CD11c^{+}; macrophages: CD45^{+} F4/80^{+}.

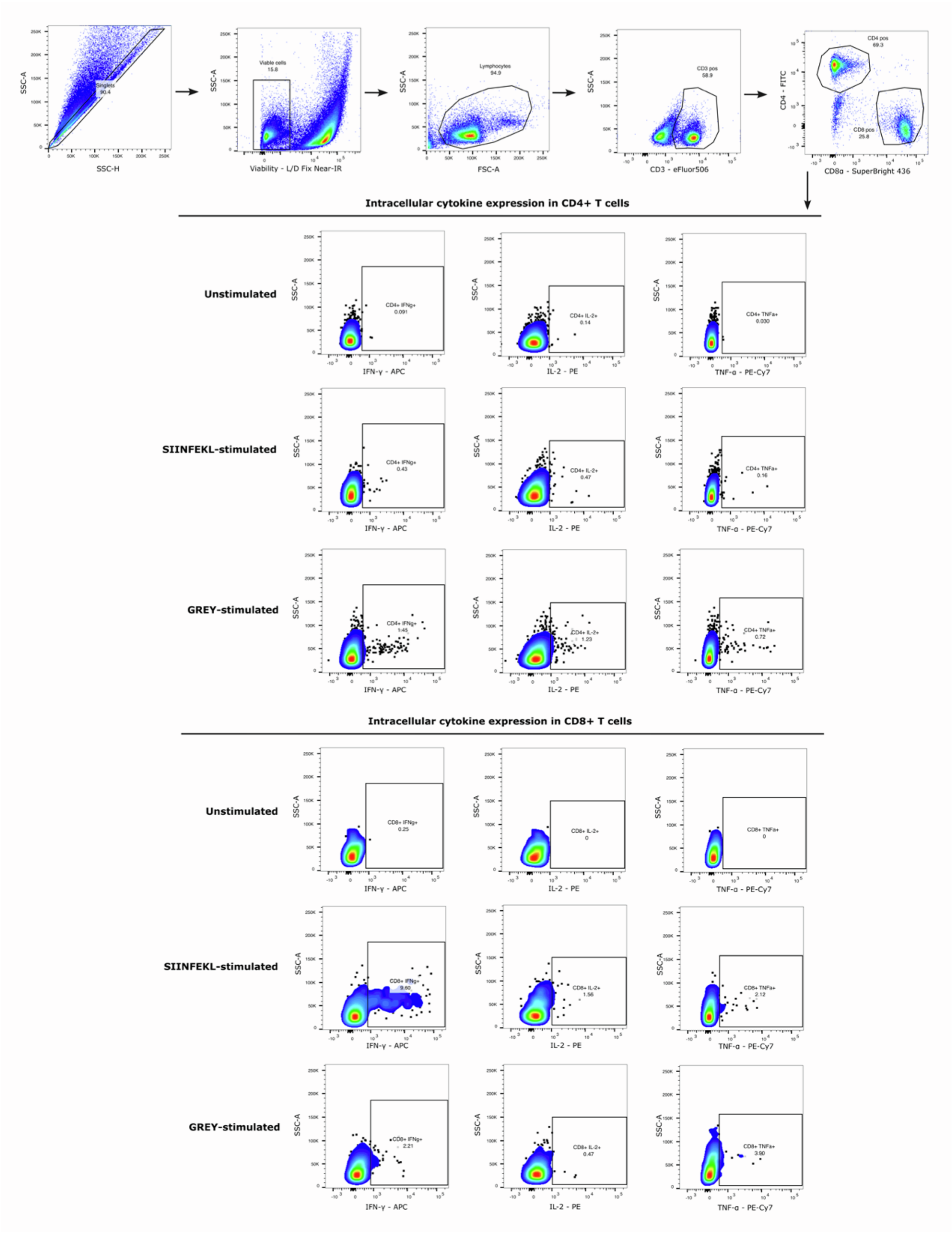

Figure S9. Gating strategy for the specific T cell activation of overnight-peptide stimulated splenocytes. Viable T cells are first gated as Lymphocytes in FSC vs SSC dot plot and then gated as $CD3^+$, following by either $CD4^+$ or $CD8^+$ T cells. Intracellular cytokine staining was performed to analyze peptide-specific $CD4^+IFN\text{-}\gamma^+$, $CD4^+IL\text{-}2^+$, $CD4^+TNF\text{-}\alpha^+$, $CD8^+IFN\text{-}\gamma^+$, $CD8^+IL\text{-}2^+$ and $CD8^+TNF\text{-}\alpha^+$ T cells.

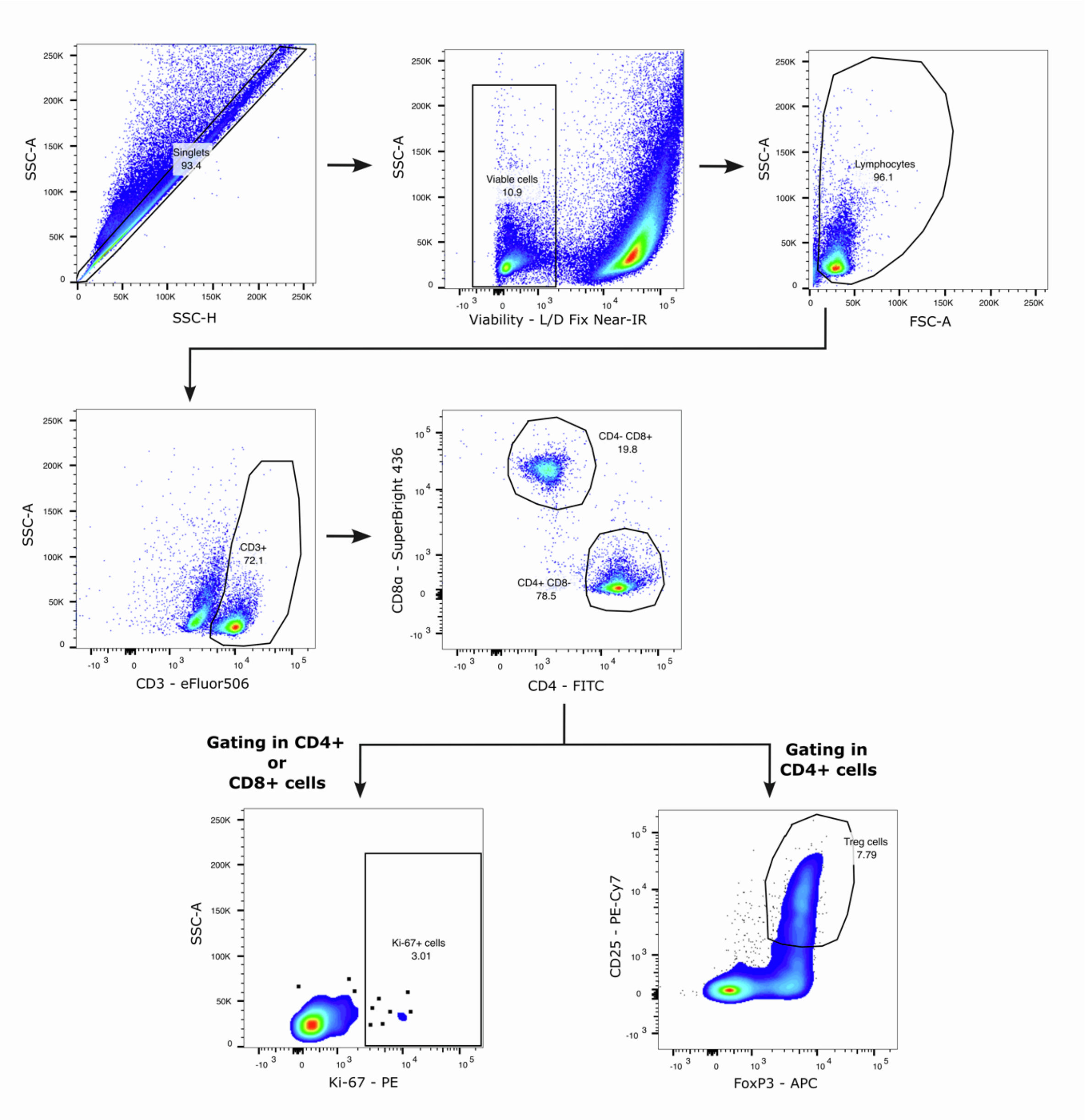

Figure S10. Gating strategy for the specific T cell proliferation and Treg response of 48h-peptide and OVA protein stimulated splenocytes. Viable T cells are first gated as Lymphocytes in FSC vs SSC dot plot and then gated as CD3$^+$, following by either CD4$^+$ or CD8$^+$ T cells. Intranuclear staining was performed to analyze the specific proliferation of CD4$^+$Ki-67$^+$ and CD8$^+$Ki-67$^+$ T cells and the proportion of Treg cells gated as CD4$^+$CD25$^+$FoxP3$^+$ T cells.
